# Efficacy and Safety of Cardioband in Patients with Tricuspid Regurgitation: Systematic Review and Meta-Analysis of Single-Arm Trials and Observational Studies

**DOI:** 10.3390/jcm13216393

**Published:** 2024-10-25

**Authors:** Eugenia Piragine, Sara Veneziano, Sabrina Trippoli, Andrea Messori, Vincenzo Calderone

**Affiliations:** 1 Department of Pharmacy, University of Pisa, 56126 Pisa, Italy; eugenia.piragine@unipi.it (E.P.); sara.veneziano@phd.unipi.it (S.V.); 2 Specialization School in Hospital Pharmacy, University of Pisa, 56126 Pisa, Italy; 3 HTA Unit, Centro Operativo, Regione Toscana, 50136 Firenze, Italy; sabrina.trippoli@estar.toscana.it (S.T.); andrea.messori@regione.toscana.it (A.M.)

**Keywords:** tricuspid regurgitation, transcatheter tricuspid valve intervention, Cardioband, systematic review, meta-analysis

## Abstract

**Background/Objectives**: The incidence and prevalence of tricuspid regurgitation (TR) are increasing worldwide. “Traditional” drug therapy with diuretics is often ineffective and the identification of new strategies, including non-pharmacological ones, is an urgent need. The aim of this study was to summarize the results on the efficacy and safety of Cardioband, one of the few approved transcatheter tricuspid valve repair systems, in patients with TR. **Methods**: Three databases (Medline, Scopus, and CENTRAL) were searched to identify clinical trials and observational studies on the efficacy (primary outcome) and safety (secondary outcome) of Cardioband. A random-effects meta-analysis was performed with R software (version 4.3.3). Survival and freedom from heart failure (HF) hospitalization were estimated with the method of reconstructing individual patient data from Kaplan–Meier curves (IPDfromKM). **Results**: Eleven studies were included in this systematic review and meta-analysis. Cardioband significantly reduced annulus diameter (−9.31 mm [95% Confidence Interval, CI: −11.47; −7.15]), vena contracta (−6.41 mm [95% CI: −8.34; −4.49]), and effective regurgitant orifice area (EROA) (−0.50 cm^2^ [95% CI: −0.72; −0.28]) in patients with TR. Cardioband reduced the severity of TR and the extent of heart failure in 91% [95% CI: 85; 97] and 63% [95% CI: 52–75] of patients, respectively. Finally, Cardioband implantation was associated with prolonged survival and freedom from HF hospitalization (80.1% and 57.8% at 24 months, respectively). **Conclusions**: This study demonstrates that Cardioband implantation leads to cardiac remodeling and mechanical improvements, reduces the severity of TR, and improves quality of life. Therefore, Cardioband is an effective option for the non-pharmacological treatment of TR.

## 1. Introduction

Tricuspid regurgitation (TR) is a valvular heart disease that affects more than 70 million people worldwide [1], with an annual incidence of about 200,000 and 300,000 cases in the United States and Europe, respectively [2]. The prevalence of TR has gradually increased over the years, especially in women, mainly due to the aging of population [3]. Congenital or acquired tricuspid valve disease is the main cause of primary TR, whereas significant dilatation of the right ventricle, tricuspid annulus, or right atrium is generally associated with secondary TR (80% of all cases) [4,5]. Another form of secondary TR is cardiac implantable electronic device (CIED)-related TR due to changes in the structure of the tricuspid valve following CIED implantation [6,7]. The staggering health and economic burden of this valvular heart disease mainly results from delays in diagnosis and treatment, leading to reduced quality of life, high rates of hospitalization, and increased risk of all-cause mortality [8,9,10,11]. In particular, the continuous right ventricular overload in patients with chronic, untreated, severe TR leads to irreversible cardiac damage and heart failure [4,8]. 

First-line treatment for patients with signs and symptoms of right heart failure (i.e., diuretics) is often ineffective [12,13,14], and surgery is associated with high risk of mortality (from 8.2% to 27.6%) [15,16,17] and complications over time [18,19]. Therefore, the identification of new strategies for the clinical management of patients with severe TR is an urgent need. In this regard, transcatheter tricuspid valve intervention (TTVI) represents an innovative approach to overcome the limitations of medical therapy and surgery [10,20]. Based on the mechanism of action, medical devices for TTVI can be classified into two main groups: transcatheter tricuspid valve repair systems and transcatheter tricuspid valve replacement systems [21,22,23]. A careful pre-procedural assessment of the morphological and functional parameters of the tricuspid valve is essential to achieve maximum TR reduction after the procedure and to avoid ineffective interventions. In particular, the transcatheter annuloplasty technique is recommended in patients with short leaflet length and severe annular dilatation, which is a hallmark of patients with severe or major TR [24,25]. The Cardioband annuloplasty system (Edwards Lifesciences, Irvine, CA, USA) is one of the few transcatheter repair devices approved in the European Union for the treatment of TR [26]. In 2018, Cardioband received the CE (Conformité Européenne) mark, which certifies that the medical device complies with European legislation [27]. Cardioband consists of a sutureless ring with a polyester fabric covering with radiopaque markers and a contraction wire inside, connected to a size adjustment system. Once inserted through a femoral venous access, it is secured along the atrial side of the tricuspid annulus with several anchors and then contracted to directly reduce dilatation of the pathologic annulus, with lower procedural risk than surgery even for inoperable patients with severe TR [20,22,26,27]. However, the few available studies on Cardioband have mainly focused on specific outcomes over short follow-up periods, thus being poorly representative of the “real-world” setting. Previous meta-analyses have demonstrated the overall efficacy and safety of all transcatheter repair devices, but they have not shown results for Cardioband alone [28,29,30,31]. In addition, other meta-analyses have focused on specific devices designed for TTVI (e.g., MitraClip or PASCAL) [32,33,34,35] but have not included Cardioband. Therefore, a comprehensive overview of the efficacy and safety of this medical device is lacking. 

The aim of this systematic review was to summarize the results on the efficacy and safety of Cardioband in patients with TR through a meta-analysis of clinical trials and observational studies. In addition, the method of reconstructed individual patient data (IPD) from Kaplan–Meier curves was applied to provide further information on the long-term efficacy of Cardioband in terms of survival and freedom from heart failure (HF) rehospitalization.

## 2. Materials and Methods

This systematic review and meta-analysis has been conducted according to the preferred reporting items for systematic reviews and meta-analysis (PRISMA) guidelines. The protocol has been registered in the PROSPERO database (CRD42024539677).

### 2.1. Search Strategy and Study Selection

The Medline (via PubMed), Scopus, and CENTRAL (via the Cochrane Library) databases were searched for studies published until 21 February 2024. The search strategy was composed of two terms combined with the Boolean operator “AND”. The first term was tricuspid regurgitation, while the second term was Cardioband (Supplementary Material S1).

The study selection was performed using the bibliographic management software Rayyan^®^ (version 1.5.1). The titles and abstracts of the identified studies were independently screened by two authors (E.P. and S.V.). Each article was considered irrelevant or potentially eligible based on the predefined inclusion and exclusion criteria. Then, the full text of potentially eligible papers was retrieved or, if not available, was requested directly from the first author. Full texts were evaluated by two authors (E.P. and S.V.), and studies that did not fit the eligibility criteria were excluded. Throughout the process, any disagreements were discussed with other authors (V.C. and A.M.), who further checked the titles, abstracts and full texts to clarify whether “controversial” papers met the inclusion criteria.

### 2.2. Inclusion Criteria

Clinical trials and observational studies on patients with TR undergoing Cardioband implantation (regardless of age, gender, comorbidities, TR etiology, and severity), written in English, were included. The primary outcome was the efficacy of Cardioband, while the secondary outcome was safety. 

### 2.3. Exclusion Criteria

Letters to the editor, studies that showed a single overall result for multiple devices, articles with non-retrievable efficacy data on Cardioband, and secondary analyses of already selected studies were excluded. Case reports and case series were also excluded, as suggested by current guidelines for systematic reviews, such as PRISMA and GRADE. In fact, these study designs usually have questionable internal validity and provide a low level of evidence [36,37].

### 2.4. Data Extraction

The following data were extracted: study design; description of the study population as reported by the authors; number and general characteristics of patients, including age, gender, comorbidities, and concomitant pharmacological treatments; severity of patients according to TR grade and New York Heart Association (NYHA) Classification of Heart Failure; length of follow-up period; efficacy outcomes; procedural and post-procedural outcomes (safety). When multiple follow-up periods were evaluated in a single study, information on each follow-up was collected. 

Data extraction was independently performed by two authors (E.P. and S.V.) using a Microsoft Excel (version 2022) spreadsheet. Any disagreement was resolved with a third author (V.C.).

### 2.5. Risk-of-Bias Assessment

The methodological quality of the included studies was independently assessed by two authors (E.P. and S.V.) using a modified version of the Newcastle–Ottawa Quality Assessment Scale (NOS) for cohort studies [38]. The tool consisted of a 4-item domain (i.e., “selection”) and a 5-item domain (i.e., “outcome”). The first domain assessed the representativeness of the target population, the absence of the outcomes of interest at baseline, the description of the study population, and the total number of patients. The second domain included an item on the adequacy of follow-up for long-term outcomes, two items on the validity of outcome measurement methods (one for functional/structural parameters and one for disease severity), and two items on follow-up rate (one for functional/structural parameters and one for disease severity). Each study was assigned a maximum score of 9. Studies were classified as “low risk of bias” for a total score between 7 and 9, “moderate risk of bias” for a total score between 4 and 6, and “high risk of bias” for a total score between 0 and 3.

### 2.6. Meta-Analysis

If at least three studies were homogeneous in terms of patient population and efficacy outcomes, a random-effects meta-analysis was performed using R software (version 4.3.3). 

For each echocardiographic parameter, values at baseline (pre-implantation) and follow-up (post-implantation) were expressed as mean ± standard deviation (SD). When data were shown as median and interquartile range (IQR), means and SD were calculated with the equations described by Hozo et al. [39]. A random-effects meta-analysis of mean differences (MD) was performed to calculate the overall MD with 95% Confidence Interval (95% CI) between pre-implantation and post-implantation. 

The change in TR severity and NYHA Functional Class after Cardioband implantation was expressed as the proportion of patients who achieved a reduction in TR severity or NYHA Functional Class out of the total number of patients “at risk” (i.e., those who were not in the lowest classes). When studies reported the percentage of patients in each TR severity grade or NYHA Functional Class at the end of follow-up, proportions were calculated. Then, a random-effects meta-analysis of proportions was performed to obtain pooled proportions with 95% CI.

In all meta-analyses, when studies reported a single follow-up period, we used the results for that follow-up. When studies reported more than one follow-up period, we analyzed the results of the most represented time point in all included studies (i.e., discharge or 30 days) for homogeneity.

Study-specific weights were estimated with the inverse variance method, while between-study variance (τ^2^) was calculated with the restricted maximum likelihood (REML) estimator. Heterogeneity was quantified with the Higgins heterogeneity index (I^2^) and was classified according to Cochrane guidelines, with slight modifications: not important (from 0% to 40%); moderate (from 41% to 60%); substantial (from 61% to 80%); considerable (from 81% to 100%) [40]. Heterogeneity was also assessed by Cochran’s Q test. Statistical significance was set at *p*-value < 0.05. When heterogeneity was substantial or considerable, meta-analyses stratified by risk of bias/echocardiographic technique or sensitivity analyses were performed to investigate the possible source of heterogeneity.

### 2.7. Evaluation of the Efficacy Outcomes over Time

For each outcome, when at least three studies reported multiple follow-up periods, changes in efficacy data over time were not meta-analyzed but shown directly in a graph. Indeed, stratification by follow-up period was not possible due to the risk of “falsification” of the number of patients in each analysis (i.e., the risk of considering the same patients several times in the same forest plot). For each follow-up period, the change from baseline was calculated as the mean ± standard error of the mean (SEM), and an XY scatterplot was created with GraphPad Prism 6.0 software.

### 2.8. Reconstruction of Individual Patient Data from Kaplan Meier Curves

Individual patient data (IPD) were reconstructed from Kaplan–Meier curves of survival and/or freedom from HF rehospitalization using the “IPDfromKM” and “survival” packages of the R software (version 4.3.3). Then, the reconstructed Kaplan–Meier curves were generated with the “survival” and “survminer” packages (R software, version 4.3.3) [41]. Heterogeneity between the reconstructed curves was assessed with the log-rank test, and once the hypothesis of proportional hazards over time was confirmed [42], a Cox proportional hazards model was applied using the “coxph” function of the R software (version 4.3.3). Comparisons were performed by calculating hazard ratios (HRs) with 95% CI. If curves were not heterogeneous (*p*-value > 0.05) and if the HRs with 95% CI were overlapping, the reconstructed IPD of each study was combined with the “rbind ()” function of the R software (version 4.3.3) to create a single data frame and generate pooled curves of survival and freedom from HF rehospitalization. Data were shown as percentages of survival and freedom from HF rehospitalization, with relative 95% CIs calculated at 6, 12 and 24 months after Cardioband implantation.

### 2.9. Procedural and Post-Procedural Outcomes Assessment (Safety)

Procedural and post-procedural outcomes reported in the included studies were extracted and data retrieved. Data expressed as medians and IQRs were converted to means ± SD as described by Hozo et al. [39]. For outcomes present in at least two studies, data were pooled and shown as mean ± SD or percentage of patients with relative range. When studies reported more than one follow-up period, results from the highest time point were considered.

## 3. Results

### 3.1. Study Selection

**Figure 1:**
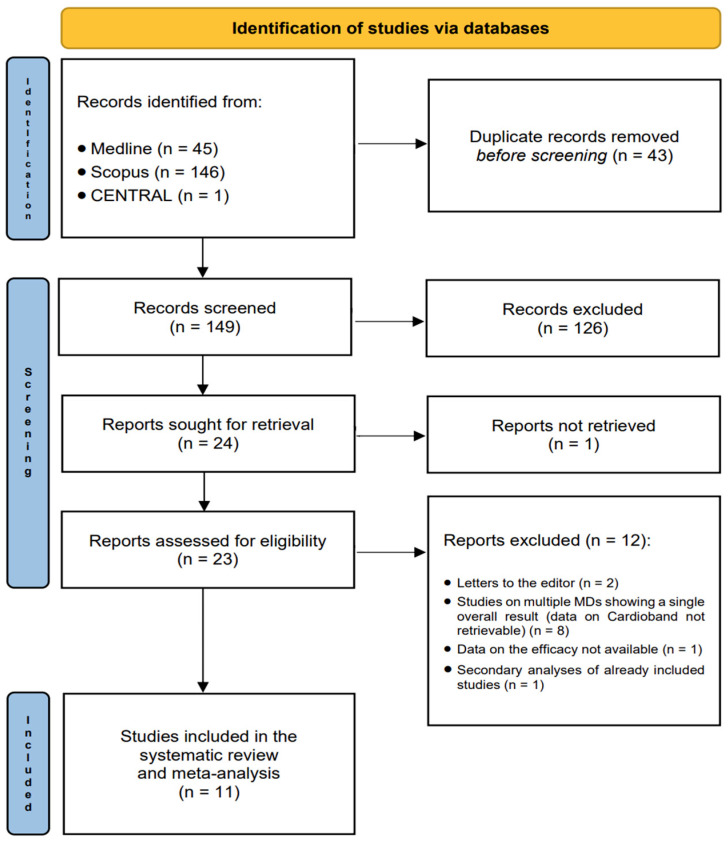
PRISMA flowchart.

Details of the study selection process are shown in Figure 1. A total of 45 studies were identified through Medline search, 146 through Scopus search, and 1 study through CENTRAL search. After eliminating duplicates, 149 records were screened and 24 were retrieved. The full text of one study was requested from the first author, who did not reply. Among the 23 records evaluated for eligibility, 12 were excluded because they were letters to the editor (2 records), studies on multiple medical devices (data on Cardioband not retrievable; 8 records), studies that did not report efficacy data (1 record), or secondary analyses of already included studies (1 record). Therefore, 11 studies were included in this systematic review and meta-analysis. 

### 3.2. Characteristics of the Included Studies

The characteristics of the studies included in this systematic review and meta-analysis are shown in Table S1. Six were single-arm trials [26,43,44,45,46,47], while five were observational studies [27,48,49,50,51]. Patients were affected by symptomatic TR [27,48]; symptomatic, chronic, and functional TR [43,44,45,46,47]; TR and acute right coronary artery deformation following Cardioband implantation [49]; and symptomatic and high-risk surgical/inoperable TR [26,50,51]. In general, most patients belonged to NYHA Functional Class III–IV (64.9–95.4%) and almost all had TR grade ≥ severe (approximately 94.0%), except for two studies [26,46] in which the percentage of patients with TR grade ≥ severe was lower (76.0%). Most patients with TR grade ≥ severe had massive or torrential TR (51.7–80.0%), although some studies did not specify this additional detail [27,50].

The number of patients ranged from 14 [49] to 74 [51] (mean: 44). The weighted mean (±SD) percentage of women was 77.2 ± 6.5%, with a weighted mean (±SD) age of 77.6 ± 1.8 years. The most common comorbidities were atrial fibrillation/flutter [26,27,43,44,45,46,47,50,51], stroke [26,27,44,45,46,49,50,51], coronary artery disease [26,27,43,44,45,46,49,50,51], heart failure [26,27,46], pulmonary hypertension [26,43,44,45,46,47], kidney dysfunction/renal disease [26,27,43,44,45,46,47], and diabetes [26,27,43,44,45,46,47,49,50,51]. In general, patients were treated with diuretics [27,43,44,45,46,47,48,50], anti-hypertensive drugs, and anticoagulants [48,50]. This cohort is similar to studies on other devices for TTVI, such as MitraClip [52,53] and TriClip [54,55], in terms of age and comorbidities. However, the proportion of women was lower (51% to 66%) in the Mitraclip and TriClip studies, while patients had a slightly lower severity of tricuspid regurgitation in the TriClip study by Nickenig and colleagues [54].

Efficacy outcomes were assessed at discharge in nine studies, [27,44,45,46,47,48,49,50,51], at 30 days in five studies [26,27,43,45,51], at 6 months in five studies [26,27,44,47,51], at 1 year in three studies [44,46,47], and at 2 years in one study [46] (some studies selected more than one follow-up period). The most reported outcomes were annulus diameter [26,27,43,44,45,46,48,49,50], effective regurgitant orifice area (EROA) [26,27,43,44,45,46,47,48], vena contracta [26,27,43,44,45,46,47,48,49], right ventricular end-diastolic diameter [26,27,43,44,45,46,49,50], right ventricular fractional area change (RVFAC) [43,45,46,47,50], systolic pulmonary artery pressure (sPAP) [26,27,43,45,46], left ventricular ejection fraction (LVEF) [26,27,43,44,45,46,50], right atrial volume [44,45,46], tricuspid annular plane systolic excursion (TAPSE) [27,44,45,46,47,49,50], TR severity reduction [26,27,43,44,45,46,47,48,49,50,51], NYHA Functional Class reduction [26,27,43,44,45,46,47,51], and Kansas City Cardiomyopathy Questionnaire (KCCQ) [26,43,44,45,46]. They were all included in the meta-analysis. 

Other outcomes were annular area [43,45,47], inferior vena cava diameter [27,43,45], 6 min walk distance [26,43,44,46,47], inferior vena contracta diameter [44], left ventricular stroke volume [26,43,45], tricuspid valve tenting height [43,44,45], coaptation gap [27,49], tricuspid ring diameter [49], tricuspid regurgitant volume [26,27], respiratory variability of the vena cava and stroke volume index [27], right atrium area [50], edema absence [26,46], hepatic vein flow reversal [45], systolic hepatic reflux [49], cardiac output and EuroQol 5-dimensions 5-level health questionnaire [45], and right ventricular ejection fraction and significant edema [47]. However, these parameters were (i) assessed in fewer than three studies; (ii) represented graphically; or (iii) not extractable. Therefore, they were not included in this meta-analysis. Regarding the most reported outcomes, annular area [43,45,47] and inferior vena cava diameter [27,43,45] were found to be significantly reduced after Cardioband implantation. In contrast, the results on 6 min walk distance were controversial: two studies [43,44] reported no change at the end of the follow-up period, while three studies showed a significant increase in this parameter [45,46,47]. Left ventricular stroke volume increased after Cardioband implantation in two [26,45] of the three studies [26,43,45]. Finally, no significant changes were observed for tricuspid valve tenting height [43,44,45]. 

Five studies reported data on survival [27,44,46,47,51] and four on freedom from heart failure rehospitalization [27,44,46,47]. However, one study did not show results with a Kaplan–Meier curve and was excluded from the analysis of reconstructed IPD [27].

### 3.3. Results of the Risk-of-Bias Assessment

**Figure 2:**
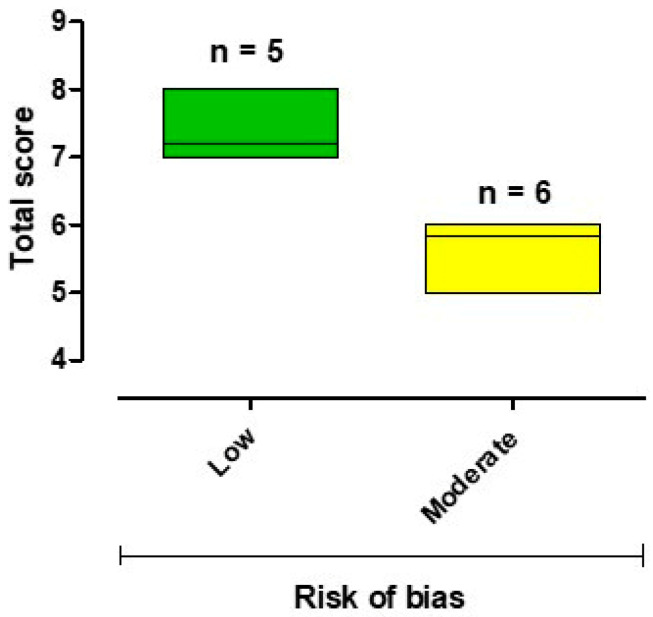
Summary of the risk-of-bias assessment.

The methodological quality of the included studies, assessed with the NOS, was acceptable. Five studies scored 7–9 and were classified at a “low” risk of bias [27,43,48,50,51], while six studies scored 4–6 and had a “moderate” risk of bias [26,44,45,46,47,49] (Figure 2 and Table S2). The “moderate” risk of bias in these studies was mainly due to population selection (e.g., inclusion of patients with right coronary artery deformation [49] or selection of few patients [26,44,46,47,49]) or measuring outcomes (e.g., studies with a short follow-up period [45,49] or too many patients lost to follow-up [26,44,45,46,47]).

### 3.4. Results of the Meta-Analysis

#### 3.4.1. Effects of Cardioband on Echocardiographic Parameters

##### Annulus Diameter

**Figure 3:**
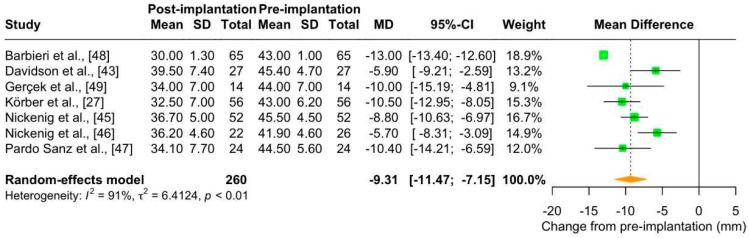
Forest plot of mean difference (MD) with 95% Confidence Interval (CI) of annulus diameter (mm) between post-implantation (follow-up) and pre-implantation (baseline) of Cardioband in patients with tricuspid regurgitation.

Patients with TR generally have an excessive diameter and flatness of the tricuspid annulus due to abnormal cardiac remodeling [56]. The results of the meta-analysis of seven studies (number of patients: 260; maximum follow-up: 30 days) showed that Cardioband implantation significantly reduced annulus diameter in patients with TR (mean change from pre-implantation: −9.31 mm [95% CI: −11.47; −7.15]) (Figure 3). This effect was clinically relevant, as the annulus diameter after Cardioband implantation was less than 42 mm (mean: 34.6 mm [95% CI: 32.23–37.01]), which is the cut-off for classifying TR as severe (Table S3).

Heterogeneity was considerable (I^2^ = 91%). To examine possible sources of heterogeneity, we investigated potential differences among studies in terms of (i) parameter value at baseline; (ii) TR severity at baseline; and iii) risk of bias. Annulus diameter at baseline was quite similar across all included studies (mean: 43.85 mm [95% CI: 42.76; 44.94]; Table S3). When we performed a sensitivity analysis by removing the single study with a high proportion of patients with less than severe TR [46], the heterogeneity remained considerable (mean change from pre-implantation: −9.99 mm [95% CI: −12.10; −7.88], I^2^ = 88%). We then stratified the results by risk of bias (Figure S1) but no significant difference between groups was found (*p*-value = 0.46). Interestingly, in the analysis stratified by the echocardiographic technique used, i.e., transesophageal echocardiography (TEE) and transthoracic echocardiography (TTE) (Figure S2), the heterogeneity was reduced to substantial (I^2^ = 61% and 62%, respectively), with significant differences between groups (*p* = 0.03). This indicates that differences in echocardiographic techniques could be a possible source of heterogeneity. In particular, the reduction in annulus diameter was greater when measured by TEE. In addition, heterogeneity was also reduced in the analysis stratified by the “phase” at which the parameter was measured (i.e., end-diastolic or not specified; I^2^ = 61% and 22%, respectively), with significant differences between groups (*p* < 0.01) (Figure S3), suggesting that this technical detail might have influenced the heterogeneity of the whole analysis.

**Figure 4:**
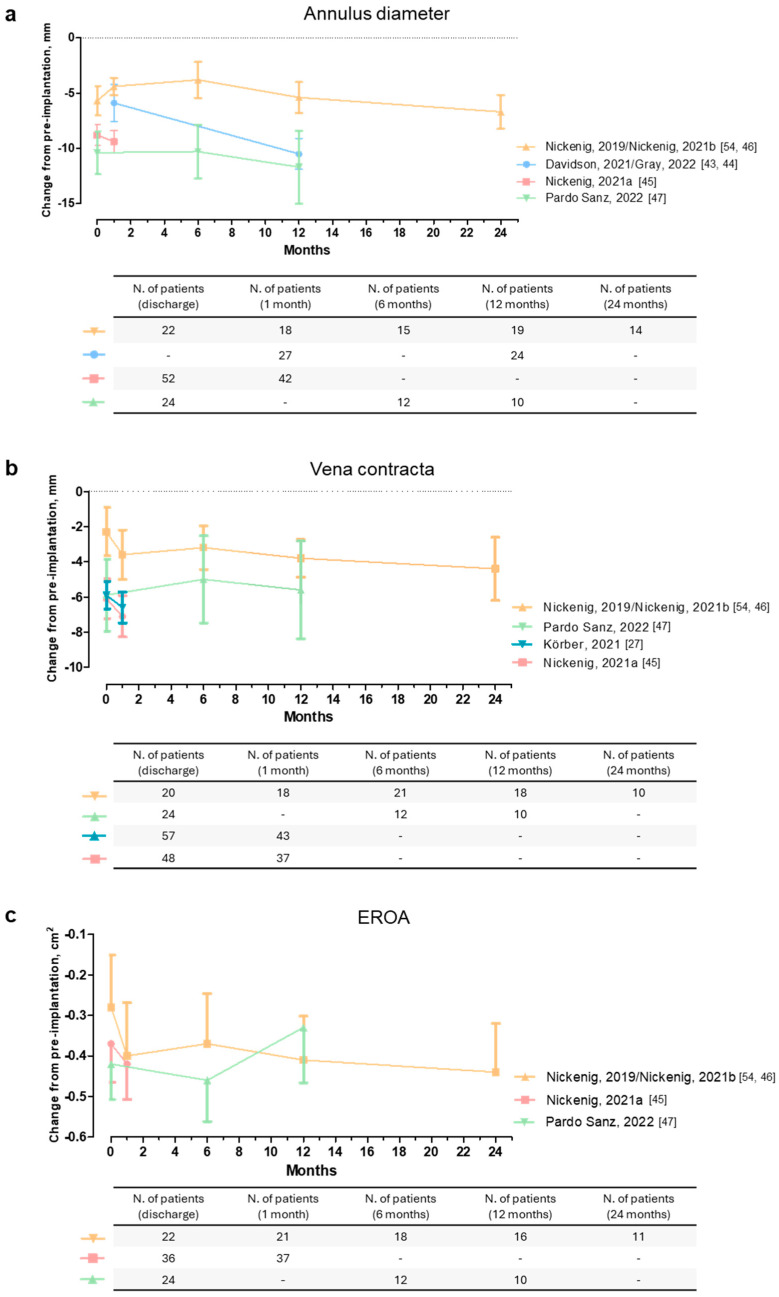
This figure shows the change in annulus diameter (**a**), vena contracta (**b**), and EROA (**c**) after Cardioband implantation in patients with TR. Data are from studies with multiple follow-up periods (0 months = at discharge) and are shown as mean ± SEM. The tables show the number of patients for each time point.

The follow-up periods were intentionally overlapped (from discharge to 30 days after Cardioband implantation) to improve the reliability of the results. In fact, we did not include multiple follow-up periods to avoid overestimating the number of patients in the same analysis. It is noteworthy that when we plotted the data from studies that reported multiple follow-up periods in a graph, we observed that the effectiveness of Cardioband in reducing annulus diameter was maintained and, in some cases, even increased over time. However, the low number of studies available (n = 4) does not allow us to provide conclusive evidence on the long-term effectiveness of Cardioband (Figure 4a).

##### Vena Contracta

**Figure 5:**
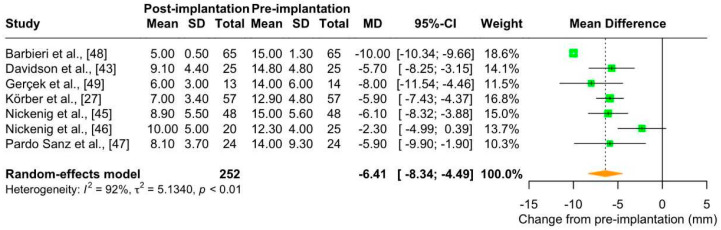
Forest plot of mean difference (MD) with 95% Confidence Interval (CI) of vena contracta (mm) between post-implantation (follow-up) and pre-implantation (baseline) of Cardioband in patients with tricuspid regurgitation.

Patients with TR have an increased vena contracta, which is defined as the diameter of a fluid flow at a point where the velocity is maximum, and the diameter is minimum [57]. The meta-analysis of seven studies (number of patients: 252; maximum follow-up: 30 days) showed a significant reduction in the vena contracta in patients with TR who underwent Cardioband implantation (mean change from pre-implantation: −6.41 mm [95% CI: −8.34; −4.49]), with considerable heterogeneity (I^2^ = 92%) (Figure 5). This effect was clinically relevant, as vena contracta after Cardioband implantation was almost halved (mean: 7.6 mm [95% CI: 6.22–8.94]) and close to the cut-off for classifying TR as moderate (i.e., 7.0 mm; Table S3). 

The possible sources of heterogeneity were investigated as in Section “Annulus Diameter”. At baseline, patients had similar characteristics in terms of vena contracta (mean: 14.05 mm [95% CI: 13.07; 15.02]; Table S3). In the sensitivity analysis performed by removing the study with a high proportion of patients with less than severe TR [46], heterogeneity remained considerable (mean change from pre-implantation: −7.14 mm [95% CI: −8.87; −5.40], I^2^ = 90%). No significant differences were found between groups in the analysis stratified by risk of bias (Figure S4) (*p*-value = 0.31). Finally, in the analysis stratified by the echocardiographic technique used, i.e., TEE and TTE (Figure S5), heterogeneity was markedly reduced (I^2^ = 18% and 49%, respectively), with significant differences between groups (*p* < 0.01). This indicates that differences in echocardiographic techniques could be a possible source of heterogeneity. In particular, vena contracta reduction was greater when measured by TEE. Again, follow-up periods were deliberately overlapping (from discharge to 30 days after Cardioband implantation). When we extracted data from studies with multiple follow-up periods, we highlighted the long-term effectiveness of Cardioband. However, the number of studies was very low (n = 4) and further investigations are needed (Figure 4b).

##### EROA

**Figure 6:**
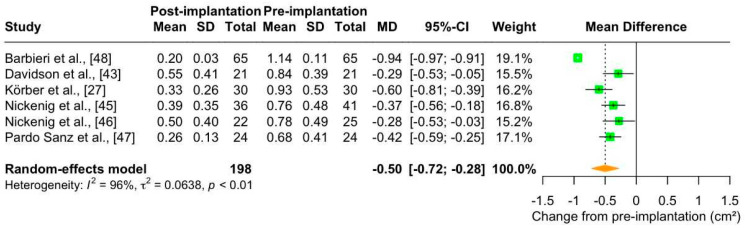
Forest plot of mean difference (MD) with 95% Confidence Interval (CI) of effective regurgitant orifice area (EROA; cm^2^) between post-implantation (follow-up) and pre-implantation (baseline) of Cardioband in patients with tricuspid regurgitation.

EROA is increased in patients with TR and is routinely measured to quantify disease severity [56,58]. The meta-analysis of six studies (number of patients: 198; maximum follow-up: 30 days) demonstrated that Cardioband implantation significantly reduced EROA in patients with TR (mean change from pre-implantation: −0.50 cm^2^ [95% CI: −0.72; −0.28]), with considerable heterogeneity (I^2^ = 96%) (Figure 6). This effect was clinically relevant, as EROA after Cardioband implantation was almost halved (mean: 0.35 cm^2^ [95% CI: 0.25–0.46]) and less than the cut-off for classifying TR as moderate (Table S3). 

The possible sources of heterogeneity were investigated as in Section “Annulus Diameter”. At baseline, EROA was quite similar in all included studies (mean: 0.87 cm^2^ [95% CI: 0.72; 1.01]; Table S3), except for one article where it was higher [48]. When we removed this study from the analysis, the heterogeneity was markedly reduced, becoming not important (mean change from pre-implantation: −0.40 cm^2^ [95% CI: −0.72; −0.28]; I^2^ = 24%). We also performed a sensitivity analysis by removing the study with a high proportion of patients with less than severe TR [46], but the heterogeneity remained considerable (mean change from pre-implantation: −0.54 cm^2^ [95% CI: −0.78; −0.30], I^2^ = 96%). This suggests that baseline EROA, but not disease severity, could influence the effectiveness of Cardioband. No significant differences were found between groups in the analysis stratified by risk of bias (Figure S6) (*p*-value = 0.21). In the analysis stratified by the echocardiographic technique used, i.e., TEE and TTE (Figure S7), heterogeneity was nullified for TTE (I^2^ = 0%) but not for TEE (I^2^ = 90%), with significant differences between groups (*p* < 0.02). However, only two studies reported echocardiographic parameters measured by TEE, and further investigation is needed. Again, the reduction in EROA was greater when measured by TEE. Finally, follow-up periods intentionally overlapped (from discharge to 30 days after Cardioband implantation). When we extracted data from studies with multiple follow-up periods, we observed that Cardioband could be effective in reducing EROA in patients with TR over time, but the low number of studies available (n = 3) suggests the need for further studies to confirm the long-term effectiveness of Cardioband (Figure 4c).

##### Right Ventricular Diameter

**Figure 7:**
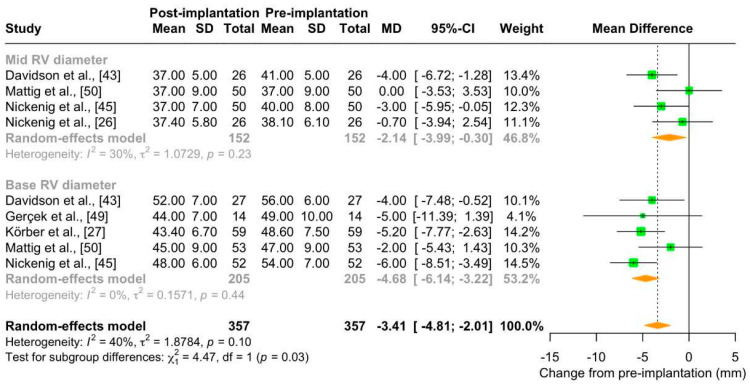
Forest plot of mean difference (MD) with 95% Confidence Interval (CI) of mid and base right ventricular (RV) diameter (mm) between post-implantation (follow-up) and pre-implantation (baseline) of Cardioband in patients with tricuspid regurgitation.

Patients with TR undergo progressive right ventricular remodeling, which can be measured as an increase in right ventricular diameter on echocardiographic examination [58]. The meta-analysis of the four studies (number of patients: 152; maximum follow-up: 30 days) that reported the measurement of mid right ventricular diameter showed a slight, but not significant, reduction in this parameter after Cardioband implantation in patients with TR (mean change from pre-implantation: −2.14 mm [95% CI: −3.99; 0.30]), with not important heterogeneity (I^2^ = 30%). The meta-analysis of the five studies (number of patients: 205; maximum follow-up: 30 days) that reported the measurement of base ventricular diameter after Cardioband implantation showed a significant reduction at the end of follow-up (mean change from pre-implantation: −4.68 mm [95% CI: −6.14; −3.22]), with no heterogeneity (I^2^ = 0%) (Figure 7). This effect was not clinically relevant, as the base right ventricular diameter after Cardioband implantation was more than 42 mm (mean: 46.50 [95% CI: 43.41–49.60), which is the cut-off for the diagnosis of TR (Table S3).

The low number of studies in each subgroup (i.e., mid and base diameter) did not allow us to perform secondary analyses stratified by risk of bias or length of follow-up period.

##### Other Parameters

The diagnosis of TR and quantification of disease severity require simultaneous measurement of numerous echocardiographic parameters. In addition to those already discussed, there are other parameters of interest that were reported in less than or equal to five studies or that did not show significant changes after Cardioband implantation. These parameters are listed in Table 1 and include TAPSE (221 patients; mean change from pre-implantation: −1.61 mm [95% CI: −3.22; 0.00]; Figure S8), LVEF (215 patients; mean change from pre-implantation: 0.02% [95% CI: −0.74; 0.78]; Figure S11), RVFAC (155 patients; mean change from pre-implantation: −2.59 [95% CI: −4.04; −1.14]; Figure S12), sPAP (163 patients; mean change from pre-implantation: 5.69 mmHg [95% CI: 2.94; 8.44]; Figure S13), and right atrial volume (99 patients; mean change from pre-implantation: −23.75 mL [95% CI: −37.75; −9.74; Figure S14). No heterogeneity was found in all analyses (I^2^ = 0%), except for TAPSE, which showed substantial heterogeneity (I^2^ = 77%). For this parameter, we stratified the results by risk of bias, but no differences were observed between subgroups (Figure S9) (*p*-value = 0.08). We also performed a sensitivity analysis by removing the study with a high proportion of patients with less than severe TR and lower TAPSE at baseline [46], but the heterogeneity remained substantial (mean change from pre-implantation: −1.92 mm [95% CI: −3.73; −0.10], I^2^ = 78%). Finally, we stratified the results according to the echocardiographic technique used, i.e., TEE and TTE (Figure S10), and found no heterogeneity for TEE (I^2^ = 0%) but substantial heterogeneity was still found for TTE (I^2^ = 75%), with significant differences between groups (*p* < 0.01). This indicates that differences in echocardiographic technique could be a possible source of heterogeneity, with the greatest heterogeneity associated with the TTE technique. Again, the reduction in TAPSE was higher when measured by TEE.

##### TR Severity

**Figure 8:**
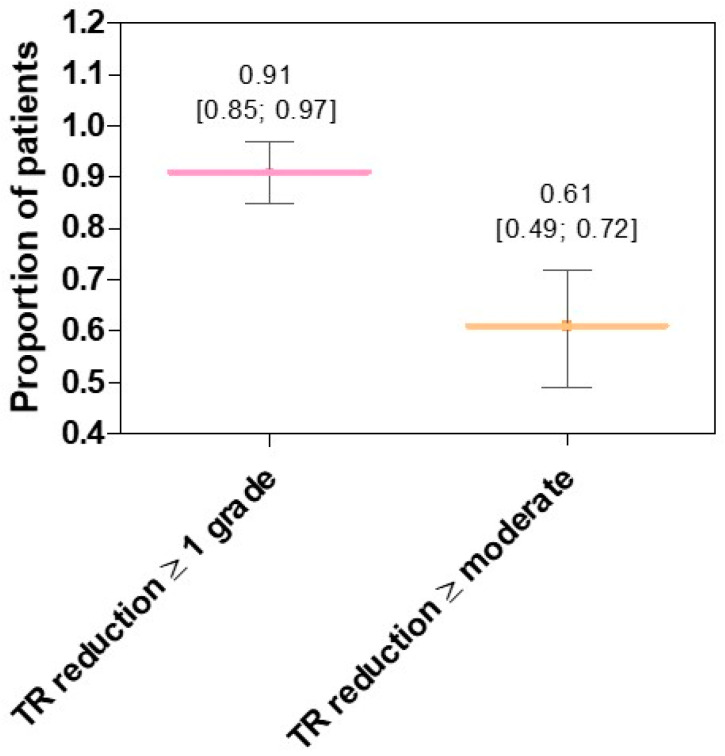
The horizontal lines show the mean proportion of patients, with 95% Confidence Interval (CI), who achieved TR reduction of at least one grade (pink line; 7 studies) and TR reduction to at least a “moderate” grade (orange line; 9 studies) after Cardioband implantation. Values are reported as mean [95% CI].

Most studies included in the systematic review also reported the number/proportion of patients who achieved a reduction in TR severity. Indeed, TR severity can be classified into five classes based on the worsening of symptoms (i.e., mild, moderate, severe, massive, and torrential) [59]. The meta-analysis of seven studies (number of patients: 354; maximum follow-up: 30 days) showed that the proportion of patients with TR reduction of at least one grade was 0.91 [95% CI: 0.85; 0.97], with considerable heterogeneity (I^2^ = 83%) (Figure 8 and Figure S15). In the analysis stratified by risk of bias (Figure S16) or echocardiographic technique, i.e., TEE and TTE (Figure S17), no differences were found between subgroups (*p*-value = 0.84 and 0.82, respectively).

The proportion of patients with TR reduction to at least “moderate” grade after Cardioband implantation was 0.61 [95% CI: 0.49; 0.72] (nine studies; 408 patients; maximum follow-up: at discharge), with considerable heterogeneity (I^2^ = 83%) (Figure 8 and Figure S18). Importantly, there were differences between subgroups in the meta-analysis on patients with TR reduction to at least “moderate” grade stratified by risk of bias (*p*-value = 0.02). In particular, studies with “low” risk of bias showed a higher proportion of patients with reduced TR severity (0.72 vs. 0.50; Figure S19). In contrast, when we stratified the results by the echocardiographic technique used, i.e., TEE and TTE (Figure S20), we found no significant differences between groups (*p* = 0.06).

#### 3.4.2. Effects on NYHA Functional Class and Self-Reported Symptoms

Six studies included in this systematic review also reported the number/proportion of patients who achieved a reduction in NYHA Functional Class after Cardioband implantation. NYHA Functional Class changes with the worsening of heart failure associated with TR (from class I to class IV) [60]. The meta-analysis of these studies (number of patients: 247; most frequent follow-up: 30 days) showed that the proportion of patients who were in NYHA Functional Class I–II at the end of the follow-up period was 0.63 [95% CI: 0.52; 0.75] (Figure S21), with substantial heterogeneity (I^2^ = 70%). In the analysis stratified by risk of bias, no differences were found between subgroups for the meta-analysis on patients who were in NYHA functional classes I–II at the end of the follow-up period (*p*-value = 0.10; Figure S22). Finally, Cardioband implantation led to an increase in self-reported patients’ perception of quality of life and heart failure symptoms as measured by the Kansas City Cardiomyopathy Questionnaire (KCCQ) [61]. Indeed, the meta-analysis of three studies (108 patients) showed that the total score increased by 16.68 points [95% CI: 10.65; 22.71] after 30 days’ follow-up (Figure S23), with no heterogeneity (I^2^ = 0%)

### 3.5. Survival and Freedom from HF Rehospitalization

**Figure 9:**
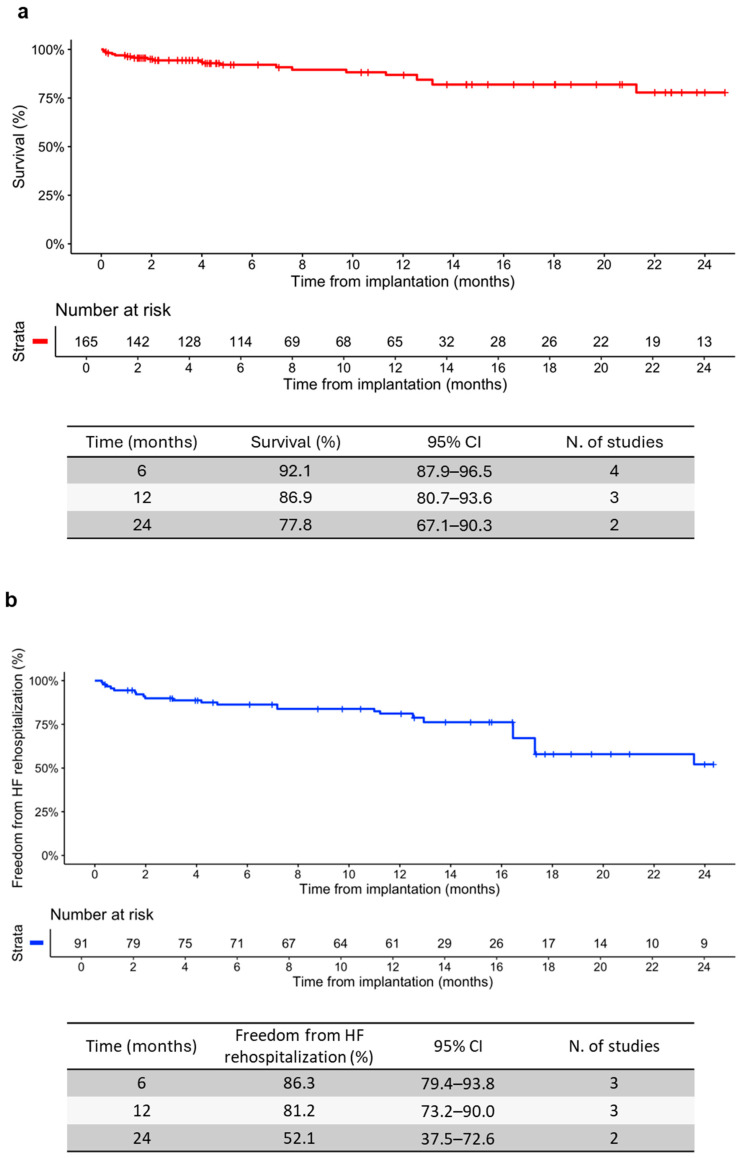
Pooled curves of survival (**a**) and freedom from heart failure (HF) rehospitalization (**b**) with the relative number of patients at risk for each time point. The tables show survival and freedom from HF rehospitalization (%), with 95% CI, and the number of studies for each time point.

IPD were reconstructed from Kaplan–Meier curves of survival (four studies; [44,46,47,51]) and freedom from HF rehospitalization (three studies; [44,46,47]), and reconstructed Kaplan–Meier curves were then generated (Figure S24). The results of the log-rank test showed no heterogeneity (*p*-value = 0.60 for survival and *p*-value = 0.33 for freedom from HF rehospitalization), and the Cox proportional hazards model confirmed the overlap of the curves (Figure S24). This allowed us to combine the reconstructed IPD to obtain a single pooled curve for survival (Figure 9a) and freedom from HF rehospitalization (Figure 9b). The aggregate analysis showed that survival was 92.1% [95% CI: 87.9–96.5] at 6 months [44,46,47,51], 88.2% [95% CI: 82.5–94.4] at 12 months [44,46,47], and 80.1% at 24 months [95% CI: 70.4–91.2] [46,47], while freedom from HF rehospitalization was 86.4% [95% CI: 79.6–93.9] at 6 months [44,46,47], 80.0% [95% CI: 71.8–89.0] at 12 months [44,46,47], and 57.8% at 24 months [95% CI: 44.9–74.4] [46,47].

### 3.6. Procedural Outcomes

The most frequent procedural outcomes reported in the included studies are shown in Table 2. The mean length of hospital stay after Cardioband implantation was 8.1 ± 7.0 days, and technical success (i.e., successful delivery and positioning of the device, as well as absence of procedural mortality) was achieved in 96.8% of patients. The most common complications during the procedure were bradycardia and occlusion of the right coronary artery in a very low percentage of patients (4.8% and 4.4%, respectively). Overall, Cardioband implantation can be considered a safe procedure.

### 3.7. Post-Procedural Outcomes

The most frequent post-procedural outcomes reported in the included studies were severe bleeding (17.5% of patients), followed by major adverse events (i.e., composite endpoint of death, myocardial infarction, need for urgent cardiothoracic surgery, stroke, pericardial effusion requiring intervention, coronary artery injury requiring percutaneous or surgical intervention, arrhythmia and conduction disorders requiring permanent pacing, new need for renal replacement therapy, severe bleeding, percutaneous or surgical non-elective tricuspid valve reintervention, major access-site and vascular complications, and major cardiac structural complications) occurred in 10.8% of patients within post-procedural 30 days or in-hospital (Table 3).

## 4. Discussion

The prevalence of TR has gradually increased over the years, posing a serious treat for the healthcare systems. “Traditional” drug therapy with diuretics is often ineffective, and TR remains undertreated. Therefore, identifying new options for the clinical management of this valvular disease is an urgent need [3,62]. An innovative and emerging strategy for the treatment of TR is TTVI, which includes transcatheter tricuspid valve repair systems and transcatheter tricuspid valve replacement systems [10]. The first CE-approved transcatheter tricuspid valve repair system is Cardioband, a high-tech, high-cost medical device that is increasingly being used in clinical practice [27]. However, there are currently few studies on the efficacy and safety of Cardioband, and a comprehensive overview is not available. 

In this systematic review and meta-analysis, we summarized the results of 11 studies on approximately 200 patients with TR. We first evaluated the effects of Cardioband on echocardiographic parameters, focusing on vena contracta, EROA, and annulus diameter, which are abnormally increased in patients with TR [56,57,58]. In fact, one of the main features of TR is annular dilation, which progressively leads to lack of leaflet coaptation and worsening of TR signs and symptoms [62]. Our results showed that Cardioband significantly reduced annulus diameter by about 9 mm in patients with TR, with an annulus diameter ≥ 40 mm at baseline in most of the included studies. This effect was clinically relevant because the annulus diameter after Cardioband implantation was less than 42 mm, which is the cut-off for classifying TR as severe. A recent meta-analysis demonstrated an inverse relationship between tricuspid annulus size and survival in patients with TR, showing a survival rate almost doubled in patients with an annulus diameter of 36 mm compared to those with a diameter of 44 mm [63]. This suggests that Cardioband, leading to a reduction in annulus diameter by about 9 mm, could effectively improve survival in patients with TR. In addition, Cardioband reduced vena contracta and EROA by about 6 mm and 0.5 cm^2^, respectively, further indicating mechanical improvements after Cardioband implantation and supporting the use of this medical device in clinical practice. These effects were clinically relevant, as the mean vena contracta after Cardioband implantation was almost halved and close to the cut-off for classifying TR as moderate, while the mean EROA after Cardioband implantation was less than 0.40 cm^2^, which is the cut-off for classifying TR as severe. 

Of note, the efficacy of Cardioband was maintained over time (up to 24 months), suggesting the long-term therapeutic potential of this high-tech medical device for the treatment of TR. However, the low number of available studies does not allow us to discuss this preliminary result further, and future research is needed. 

Cardioband also reduced RVFAC (−2.6%) and right atrial volume (−24 mL), which are generally increased in patients with TR due to cardiac dilatation and systolic disfunction [58]. This result indicates positive cardiac remodeling after Cardioband implantation and, albeit indirectly, suggests a potential better prognosis. In fact, lower RVFAC and right atrial volume were associated with a higher survival rate in patients with TR [63,64]. All these data were quite superimposable with those of studies on patients with similar characteristics at baseline who underwent implantation of other devices for TTVI (i.e., MitraClip and TriClip [52,53,54,55]), as shown in the indirect comparison we performed (Table S4).

Finally, Cardioband led to a significant increase in sPAP (~ 5.6 mmHg), thus indicating a potential risk of pulmonary hypertension in device users. However, current guidelines recommend the use of peak tricuspid regurgitation velocity (TRV), instead of sPAP, for the prediction of pulmonary hypertension [65], while some authors have suggested the use of the TRV/TAPSE ratio [66]. Therefore, future studies are needed to confirm these preliminary findings, which have not emerged in articles on other devices for TTVI (Table S4).

Notably, most of the analyses were characterized by considerable heterogeneity. When we stratified the results by risk of bias or performed sensitivity analyses by removing studies on patients with different baseline characteristics (e.g., less severe TR), we did not find a clear source of heterogeneity. On the contrary, when we stratified them according to the echocardiographic technique used, we observed a marked reduction in heterogeneity and, in particular, a greater reduction in some echocardiographic parameters (e.g., annulus diameter, vena contracta, EROA and TAPSE) measured by TEE rather than TTE, suggesting potential differences in effect size measurement between these techniques. However, at present, it is not possible to determine whether TTE, recommended by current guidelines, may underestimate—or whether TEE may overestimate—the “true” difference between baseline and post-implantation, and future studies are needed. Another source of heterogeneity could certainly be the “phase” at which the parameter is measured (i.e., early or late systole/diastole). However, most articles did not report this technical detail, except for annulus diameter. In the analysis stratified by “phase” (i.e., end-diastolic septolateral annulus diameter or not specified), we found a marked reduction in heterogeneity, suggesting that this technical detail might have influenced the heterogeneity of the whole analysis on this parameter.

The echocardiographic improvements observed after Cardioband implantation were accompanied by positive effects on clinical parameters and quality of life indicators. It is now well established that patients with higher severity of TR [67,68] and NYHA Functional Class [69] have an increased risk of hospitalization and mortality. Therefore, slowing disease progression and improving quality of life are two main goals of the TR treatment [61]. The results of our meta-analysis showed that Cardioband significantly reduced TR severity and NYHA Functional Class in 91% and 63% of patients, respectively. In addition, Cardioband implantation led to an increase in KCCQ score of about 17 points, thus indicating an improvement in symptoms and quality of life in patients with TR. In fact, lower scores represent more severe symptoms, while higher scores (up to 100) indicate optimal quality of life. As variations of 10–20 points are considered clinically significant [61], Cardioband has been shown to achieve both goals of TR therapy. It is worth noting that in the comparative study by Ochs and colleagues [51], there was a higher percentage of patients with TR reduction of ≥2 grades and ≥3 grades in the Cardioband group compared to the TEER group (66.2% vs. 58.6% for ≥2 grades and 32.4% vs. 18.4% for ≥3 grades), which included the MitraClip and TriClip systems. This suggests a potential higher effectiveness of Cardioband in reducing TR severity, but further comparative studies are needed to confirm this finding.

In addition to the “classical” meta-analysis, in this study, we also applied the innovative IPDfromKM method to summarize the effects of Cardioband on survival and freedom from HF hospitalization. Among the studies included in the meta-analysis, we selected the few that reported KM graphs. The reconstructed curves showed that Cardioband implantation was associated with sustained survival and freedom from HF hospitalization over time (about 80.1% and 57.8% at 24 months, respectively). This result is almost superimposable to that obtained after the implantation of TriClip, a transcatheter edge-to-edge repair (TEER) system, in patients with severe TR or higher. In fact, von Bardeleben and colleagues demonstrated that 2-year survival and freedom from HF hospitalization after TriClip implantation were 81% and 63%, respectively [70]. In a randomized controlled trial by Hahn and colleagues, 2-year survival was 65.9% after the implantation of Mitraclip, another TEER system, and 36.4% after the initiation of drug therapy in patients with moderate to severe TR. In addition, 2-year freedom from HF hospitalization was 62.0% after Mitraclip implantation and 26.5% after initiation of drug therapy [71]. Therefore, our results confirm the long-term efficacy of Cardioband in patients with TR and, although indirectly, suggest better clinical outcomes after Cardioband implantation, compared to drug therapy and other transcatheter systems (e.g., Mitraclip), in terms of survival and risk of hospitalization. However, the main limitation of our work is the lack of a “direct” control group, which does not allow us to provide conclusive results on the superiority or non-inferiority of Cardioband over drug therapy and other medical devices.

Finally, safety evaluation showed that technical and device success was achieved in about 97% of patients, with an average length of hospital stay of 8 days. The most common complications during the procedure were bradycardia and right coronary artery occlusion in a very low percentage of patients (~4%), while the post-procedural outcomes were mainly hemorrhagic complications (~18% of patients). Currently, there are no comparative studies on the safety of Cardioband and other annuloplasty systems proposed for TTVI. A 30-day clinical trial on TriAlign (PTVAS) reported no risk of bleeding complications in patients with moderate/severe TR undergoing medical device implantation [72]. However, only 15 patients were enrolled in this prospective study, and a 60-patient clinical trial [73] is ongoing to examine the safety and performance of TriAlign. K-Clip™ is another TTVI annuloplasty system proposed for the treatment of TR, whose efficacy was recently evaluated in a first-in-human study in patients with severe symptomatic tricuspid regurgitation [74]. No serious bleeding complications were reported in this study, as only one patient experienced mild bleeding at the transjugular access site. Again, this cohort was very small (15 patients) and further studies are needed. Regarding other categories of medical devices for TTVI, transcatheter edge-to-edge tricuspid repair (TEER) systems are the most investigated. To date, there is only one study comparing the efficacy and safety of Cardioband annuloplasty system and TEER devices [51]. In this paper, which was included in our meta-analysis, an increased risk of major or serious bleeding was reported in patients undergoing Cardioband implantation compared with TEER implantation (20.3% vs. 9.2%, respectively). Importantly, fatal bleeding did not occur in either group. This “worrying” trend was not confirmed by indirect comparisons, as the percentages of patients with TR who experienced major/serious bleeding were almost overlapping among the medical devices considered, ranging from 5.0% to 6.0% for Cardioband, from 5% to 11% for MitraClip [52,53], and from 7.2% to 11.9% for TriClip [54,55]. This highlights the discrepancies between the indirect comparisons and the results of head-to-head studies, suggesting that future studies are needed to confirm not only the efficacy but also the safety of Cardioband, and identify potential differences between genders, ages and other factors. In this regard, an interesting study by Fortmeier and colleagues recently demonstrated that procedural success, i.e., the successful implantation of a device designed for TTVI and the retraction of the delivery system with a reduction in TR of at least one grade and/or a residual TR grade ≤ II or V before discharge, is comparable between men and women with severe TR after the implantation of transcatheter systems, including Cardioband. In addition, no differences were observed between the male and female gender in 2-year survival [75]. However, our work did not address potential sex-related differences in procedural outcomes, as none of the included studies explored this intriguing aspect.

## 5. Conclusions

This meta-analysis of 11 studies showed that Cardioband has favorable and long-term outcomes in patients with TR, mainly severe-grade or higher. In particular, Cardioband implantation led to mechanical improvements, reduced cardiac remodeling, slowed disease progression and improved quality of life. Cardioband use was also associated with high survival and low HF hospitalization in the first 24 months after implantation. Finally, Cardioband demonstrated a favorable safety profile. This is the first meta-analysis summarizing the efficacy and safety of Cardioband in patients with TR, thus providing an overview of the clinical potential of this high-tech medical device. However, only single-arm trials and observational studies were included because of the lack of controlled clinical trials, which are considered the “gold standard” for efficacy evaluations, and all comparisons were made against baseline and not against a control group. Therefore, comparative studies with longer follow-up periods are needed to confirm the results of our meta-analysis, to provide conclusive findings on the efficacy and safety of Cardioband, to investigate the superiority or non-inferiority of Cardioband over drug therapy or other valvular repair/replacement systems over time, and to examine potential differences in procedural and efficacy outcomes between genders. This could guide cardiologists in choosing the most appropriate and effective medical device for the treatment of TR, as well as hospital pharmacists in monitoring health expenditure. Indeed, in many countries, hospital pharmacists are directly involved in managing medical device expenditure, thus playing a crucial role in healthcare systems.

## Figures and Tables

**Table 1:** Results of the meta-analysis on other echocardiographic parameters. List of abbreviations: CI, Confidence Interval; LVEF, left ventricular ejection fraction; MD, mean difference between post-implantation (follow-up) and pre-implantation (baseline); RVFAC, right ventricular fractional area change; sPAP, systolic pulmonary artery pressure; TAPSE, tricuspid annular plane systolic excursion.

Parameter	Maximum Follow-Up	MD	95% CI	I^2^ (%)	N. of Studies	N. of Patients	*p*-Value
TAPSE (mm)	1 year	−1.61	−3.22; 0.00	77	6	221	<0.01
LVEF (%)	30 days	0.02	−0.74; 0.78	0	5	215	0.90
RVFAC (%)	1 year	−2.59	−4.04; −1.14	0	5	155	0.56
sPAP (mmHg)	30 days	5.69	2.94; 8.44	0	4	163	0.45
Right atrial volume (mL)	1 year	−23.75	−37.75; −9.74	0	3	99	0.51

**Table 2:** Procedural outcomes reported in the studies included in this systematic review. Each patient may have experienced more than one outcome. List of abbreviations: IQR, interquartile range; SD, standard deviation.

Outcome	% of Patients (Range)	Mean ± SD or Median [IQR]	N. of Studies	Study Reference
Procedural details				
Technical success	96.8 (91.7–100.0)	-	4	[26,27,47,51]
Device success	96.3 (91.9–98.5)	-	3	[44,45,48]
Procedural success	64.8 (45.0–83.9)	-	3	[27,44,45]
Skin incision-to- femoral vein closure (min)	-	240.4 ± 72.3	4	[26,27,43,45]
Procedure time (min)	-	198.9 ± 34.8	3	[47,50,51]
Cut-to-suture time (min)		169 [144–230]	1	[48]
Fluoroscopy time (min)	-	71.6 ± 26.0	3	[43,45,48]
Contrast medium volume (mL)	-	118.8 ± 59	3	[27,50,51]
Length of hospital stay (days)	-	8.1 ± 7.0	7	[26,27,44,45,47,49,51]
Others ^1^	-	-	12	-
Complications during the procedure				
Bradycardia	4.8 (1.6–6.3)	-	3	[45,48,50]
Occlusion of right coronary artery	4.4 (1.6–8.3)	-	4	[26,27,43,45]
Right coronary artery perforation	3.3 (1.6–5.0)	-	3	[26,27,45]
Stenosis or perforation of right coronary artery	1.9 (3.3–4.8)	-	3	[26,48,50]
Right coronary artery dissection	1.6 (1.5–1.7)	-	3	[27,48,50]
Death	0.5 (0.0–3.3)	-	4	[26,47,48,50]
Others ^2^	-	-	18	-

^1^ Implant delivery system insertion-to-removal (163.1 ± 64.7 min [44,45]), number of anchors (16.0 ± 1.0 [26,27]), patients discharged home (85.6%; 80.0–88.3 [43,45]), technical and device success (98.4% [50]), radiation exposure (67.9 [45.7–123.9] gray/cm^2^ [48]), radiation exposure (61.0 [44.0–74.0] min [50]), trans-tricuspid gradient (1.5 ± 0.7 mmHg [51]), length of stay in intensive care unit (2.0 ± 1.8 days [26]), patients discharged to a rehabilitation facility or different department in the same hospital (11.7% [45]). ^2^ Major vascular access site complication (3/158; 1.9% [43,48,50]), right coronary artery stent implantation (8/90; 8.9% [26,27]), cardiac arrest necessitating cardiopulmonary resuscitation (2/129; 1.6% [48,50]), complications of right coronary artery (9/60; 15.0% [27]), worsening of pre-existing lesions of right coronary artery (1/30; 3.3% [26]), anchor disengagements (2/30; 6.7% [44]), complications in anchors’ implantation (2/24; 8.3% [47]), partial deployment of anchors (1/60; 1.7% [45]), poor transthoracic echocardiography visibility (1/60; 1.7% [45]), inability to advance the device (1/37; 2.7% [44]), cinching wire rupture (1/37; 2.7% [44]), dissection of the left iliac artery (1/63; 1.6% [50]), pericardial effusion (17/63; 27.0% [50]), not specified (29/30; 96.7% [26]).

**Table 3:** Post-procedural outcomes reported in the studies included in the systematic review. Each patient may have experienced more than one outcome.

Outcome	% of Patients (Range)	Follow-Up (Study Reference)
Bleeding complications Major Extensive Life threatening Fatal	17.5 (4.2–35.1) 8.2 (5.0–10.8) 6.1 (5.0–6.8) 2.5 (1.7–3.3) 1.5 (0.0–8.1)	30 days [27,45,51]; 1 year [44]; 2 years [46]; 279 ± 246 days [47] 30 days [27,51] 30 days [26,27,51] 30 days [26,27,51]; 1 year [44] 30 days [26,27,45,51]; 1 year [44]
Major adverse events	10.8 (6.7–19.7)	30 days [27,45,51]
All-cause mortality	7.6 (0.0–26.7)	30 days [27,45]; 6 months [26,51]; 1 year [44]; 2 years [46]; 279 ± 246 days [47]
Renal failure	6.3 (0.0–9.5)	30 days [51]; 2 years [46]; 279 ± 246 days [47]
Conduction system disturbance	3.7 (0.0–6.7)	30 days [45]; 1 year [44]; 2 years [46]; 279 ± 246 days [47]
Cardiac tamponade	3.1 (2.7–3.3)	30 days [26,27]; 1 year [44]
Stroke	1.7 (0.0–6.7)	30 days [27,45,51]; 1 year [44]; 2 years [46]; 279 ± 246 days [47]
Device-related cardiac surgery	1.6 (0.0–4.2)	30 days [27,51]; 2 years [46]; 279 ± 246 days [47]
Myocardial infarction	1.0 (0.0–1.7)	30 days [27,45,51]; 1 year [44]; 2 years [46]; 279 ± 246 days [47]
Others ^1^	-	-

^1^ Others: Major access site and vascular complications (7.1%; 6.6–8.1 at 30 days and 1 year [44,45]), clinical success (56.8%; 54.1–59.5 at 30 days [44,45]), acute renal failure (7.5%; 4.1–11.7 at 30 days [27,51]), heart failure hospitalization (8.0%; 5.9–10.8 at 30 days and 1 year [27,44]), coronary complications (7.4%; 4.2–10.0 at 279 ± 246 days and 2 years [46,47]), right coronary artery perforation (1.0%; 0.0–1.6 at 1 year and 30 days [44,45]), renal replacement therapy (2.0%; 0.0–3.3 at 1 year and 30 days [44,45]), device-related secondary intervention (5.6%; 4.2–6.7 at 279 ± 246 days and 2 years [46,47]), reintervention at tricuspid valve (0.7%; 0.0–1.4 at 30 days [45,51]), ventricular arrhythmia (5.6%; 0.0–10.0 at 1 year and 2 years [46,47]), blood transfusion (20.1%; 20.0–20.3 at 30 days [27,51]), coronary artery injury requiring intervention (6.6% at 30 days [45]), right coronary artery occlusion (3.3% at 30 days [45]), right coronary artery flow restriction (1.6% at 30 days [45]), right coronary artery temporary deformation with complete resolution (20.0% at 30 days [27]), reintervention on the previously implanted study device (5.4% at 1 year [44]), vascular complications (4.2% at 279 ± 246 days [47]), access site complication (6.8% at 30 days [51]), hemodynamically relevant arrhythmia (11.7% at 30 days [27]), need for pacemaker (3.3% at 30 days [27]), urgent open heart surgery (3.3% at 30 days [27]), uraemic encephalopathy (1.6% at 30 days [45]), right coronary artery stent implantation (3.3% at 30 days [45]), acute kidney injury (1.3% at 30 days [45]).

## Data Availability

Data are available on request from the corresponding author.

## Supplementary Materials

The following supporting information can be downloaded at https://www.mdpi.com/article/10.3390/jcm13216393/s1, Supplementary Material S1: Search strategy; Table S1: Characteristics of the included studies; Table S2: Details of the risk-of-bias assessment; Table S3: Summary of the effects of Cardioband implantation on echocardiographic parameters; Table S4: Indirect comparison of the results of our meta-analysis on Cardioband with those of other studies on medical devices for transcatheter tricuspid valve intervention (TTVI). Figure S1: Forest plot of mean difference (MD) with 95% Confidence Interval (CI) of annulus diameter (mm) between post-implantation and pre-implantation of Cardioband in patients with tricuspid regurgitation, stratified by risk of bias; Figure S2: Forest plot of mean difference (MD) with 95% Confidence Interval (CI) of annulus diameter (mm) between post-implantation and pre-implantation of Cardioband in patients with tricuspid regurgitation, stratified by echocardiographic technique (TEE: transesophageal echocardiography; TTE: transthoracic echocardiography); Figure S3: Forest plot of mean difference (MD) with 95% Confidence Interval (CI) of annulus diameter (mm) between post-implantation and pre-implantation of Cardioband in patients with tricuspid regurgitation, stratified by the “phase” at which the parameter was measured (i.e., end-diastolic septolateral annulus diameter or not specified); Figure S4: Forest plot of mean difference (MD) with 95% Confidence Interval (CI) of vena contracta (mm) between post-implantation and pre-implantation of Cardioband in patients with tricuspid regurgitation, stratified by risk of bias; Figure S5: Forest plot of mean difference (MD) with 95% Confidence Interval (CI) of vena contracta (mm) between post-implantation and pre-implantation of Cardioband in patients with tricuspid regurgitation, stratified by echocardiographic technique (TEE: transesophageal echocardiography; TTE: transthoracic echocardiography); Figure S6: Forest plot of mean difference (MD) with 95% Confidence Interval (CI) of effective regurgitant orifice area (EROA; cm^2^) between post-implantation and pre-implantation of Cardioband in patients with tricuspid regurgitation, stratified by risk of bias; Figure S7: Forest plot of mean difference (MD) with 95% Confidence Interval (CI) of effective regurgitant orifice area (EROA; cm^2^) between post-implantation and pre-implantation of Cardioband in patients with tricuspid regurgitation, stratified by echocardiographic technique (TEE: transesophageal echocardiography; TTE: transthoracic echocardiography); Figure S8: Forest plot of mean difference (MD) with 95% Confidence Interval (CI) of tricuspid annular plane systolic excursion (TAPSE; mm) between post-implantation and pre-implantation of Cardioband in patients with tricuspid regurgitation; Figure S9: Forest plot of mean difference (MD) with 95% Confidence Interval (CI) of tricuspid annular plane systolic excursion (TAPSE; mm) between post-implantation and pre-implantation of Cardioband in patients with tricuspid regurgitation, stratified by risk of bias; Figure S10: Forest plot of mean difference (MD) with 95% Confidence Interval (CI) of tricuspid annular plane systolic excursion (TAPSE; mm) between post-implantation and pre-implantation of Cardioband in patients with tricuspid regurgitation, stratified by echocardiographic technique (TEE: transesophageal echocardiography; TTE: transthoracic echocardiography); Figure S11: Forest plot of mean difference (MD) with 95% Confidence Interval (CI) of left ventricular ejection fraction (LVEF; %) between post-implantation and pre-implantation of Cardioband in patients with tricuspid regurgitation; Figure S12: Forest plot of mean difference (MD) with 95% Confidence Interval (CI) of right ventricular fractional area change (RVFAC; %) between post-implantation and pre-implantation of Cardioband in patients with tricuspid regurgitation; Figure S13: Forest plot of mean difference (MD) with 95% Confidence Interval (CI) of systolic pulmonary artery pressure (sPAP; mmHg) between post-implantation and pre-implantation of Cardioband in patients with tricuspid regurgitation; Figure S14: Forest plot of mean difference (MD) with 95% Confidence Interval (CI) of right atrial volume (ml) between post-implantation and pre-implantation of Cardioband in patients with tricuspid regurgitation; Figure S15: Forest plot of proportions, with 95% Confidence Interval (CI), of patients with tricuspid regurgitation reduction of at least one grade after Cardioband implantation; Figure S16: Forest plot of proportions, with 95% Confidence Interval (CI), of patients with tricuspid regurgitation reduction of at least one grade after Cardioband implantation, stratified by risk of bias; Figure S17: Forest plot of proportions, with 95% Confidence Interval (CI), of patients with tricuspid regurgitation reduction of at least one grade after Cardioband implantation, stratified by echocardiographic technique (TEE: transesophageal echocardiography; TTE: transthoracic echocardiography); Figure S18: Forest plot of proportions, with 95% Confidence Interval (CI), of patients with tricuspid regurgitation reduction to at least moderate-grade after Cardioband implantation; Figure S19: Forest plot of proportions, with 95% Confidence Interval (CI), of patients with tricuspid regurgitation reduction to at least moderate-grade after Cardioband implantation, stratified by risk of bias; Figure S20: Forest plot of proportions, with 95% Confidence Interval (CI), of patients with tricuspid regurgitation reduction to at least moderate-grade after Cardioband implantation, stratified by echocardiographic technique (TEE: transesophageal echocardiography; TTE: transthoracic echocardiography); Figure S21: Forest plot of proportions, with 95% Confidence Interval (CI), of patients in New York Heart Association (NYHA) Functional Class I or II after Cardioband implantation; Figure S22: Forest plot of proportions, with 95% Confidence Interval (CI), of patients in New York Heart Association (NYHA) Functional Class I or II after Cardioband implantation, stratified by risk of bias; Figure S23: Forest plot of mean difference (MD) with 95% Confidence Interval (CI) of Kansas City Cardiomiopathy Questionnaire (KCCQ) score between post-implantation and pre-implantation of Cardioband in patients with tricuspid regurgitation; Figure S24: Survival (a) and freedom from heart failure (HF) rehospitalization (b) curves of reconstructed individual patient data (IPD). Forest plots show the hazard ratios (HRs), with 95% Confidence Interval (CI), calculated with the Cox proportional hazards model.
