# Supplementary material for: Efficacy and Safety of Cardioband in Patients with Tricuspid Regurgitation: Systematic Review and Meta-Analysis of Single-Arm Trials and Observational Studies
Source: J Clin Med. 2024 Oct 25;13(21):6393. doi: 10.3390/jcm13216393 (PMC11546409; doi:10.3390/jcm13216393)
Supplement: Supplementary file 1 [file jcm-13-06393-s001.zip › jcm-3237147-supplementary.pdf]

Supplementary Material S1. Search strategy.

Medline

((((Tricuspid regurgitation) OR (tricuspid insufficiency)) OR (tricuspid valve incompetence)) OR (tricuspid valve regurgitation)) OR (tricuspid incompetence) AND ((((cardioband) OR (cardioband system)) OR (cardioband tricuspid valve reconstruction system)) OR (cardioband tricuspid system)) OR (cardioband implant)

Scopus

(TITLE-ABS-KEY (tricuspid AND regurgitation) OR TITLE-ABS-KEY (tricuspid AND insufficiency) OR TITLE-ABS-KEY (tricuspid AND valve AND incompetence) OR TITLE-ABS-KEY (tricuspid AND valve AND regurgitation) OR TITLE-ABS-KEY (tricuspid AND incompetence) AND TITLE-ABS-KEY (cardioband) OR TITLE-ABS-KEY (cardioband AND system) OR TITLE-ABS-KEY (cardioband AND tricuspid AND valve AND reconstruction AND system) OR TITLE-ABS-KEY (cardioband AND tricuspid AND system) OR TITLE-ABS-KEY (cardioband AND implant))

CENTRAL

(Tricuspid regurgitation OR tricuspid insufficiency OR tricuspid valve incompetence OR tricuspid valve regurgitation OR tricuspid incompetence) AND (cardioband OR cardioband system OR cardioband tricuspid valve reconstruction system OR cardioband tricuspid system OR cardioband implant) in Title Abstract Keyword

Table S1. **Characteristics of the included studies.** List of abbreviations: 6-MWD, 6-min walk distance; AA, annular area; ACEi, angiotensin-converting enzyme inhibitors; AD, annulus diameter; ARBs, angiotensin II receptor antagonists; ARNIs, angiotensin receptor-neprilysin inhibitors; CG, coaptation gap; CO, cardiac output; COPD, chronic obstructive pulmonary disease; EA, edema absence; EQ-5D-5L, EuroQol 5-dimensions 5-level health questionnaire; EROA, effective regurgitant orifice area; HVFR, hepatic vein flow reversal; IQR, interquartile range; IVC, inferior vena cava; KCCQ, Kansas City Cardiomyopathy Questionnaire; LVEF, left ventricular ejection fraction; LVSV, left ventricular stroke volume; NOACs, non-vitamin K antagonist oral anticoagulants; NYHA, New York Heart Association; RAA, right atrium area; RAV, right atrial volume; RCA, right coronary artery; RV, right ventricular; RVEF, right ventricular ejection fraction; RVFAC, right ventricular fractional area change; RVVC, respiratory variability of the vena cava; SD, standard deviation; SE, significant edema; SGLT-2i, sodium-glucose cotransporter-2 inhibitors; sHR, systolic hepatic reflux; sPAP, systolic pulmonary artery pressure; SVI, stroke volume index; TAPSE, tricuspid annular plane systolic excursion; TR, tricuspid regurgitation; TRD, tricuspid ring diameter; TRV, tricuspid regurgitant volume; TVTH, tricuspid valve tenting height; VC, vena contracta. Symbols: * calculated on 36/37 patients; ** calculated on 25/30 patients. *** calculated on 53/61 patients; ^ mean ± SD.

Study name	Study design	Study population	N. of patients (women, %)	Age, years (mean ± SD or median [IQR])	TR grade, %	NYHA Functional Class, %	Comorbidities, %	Drugs, %	Follow-up	Efficacy outcomes
Barbieri, 2023	Observational study	Patients with symptomatic TR	65 (63.1)	80 [77 - 83]	Less than severe (2.5); severe (18.5); massive (38.5); torrential (41.5)	II (18.5); III (75.4); IV (6.1)	-	Loop diuretics (93.8); beta-blockers (89.2); ACEi/ARBs/ARNIs (75.4); aldosterone antagonists (56.9); thiazides (26.2); SGLT-2i (24.6)	Discharge	AD; EROA; VC; TR severity reduction
Davidson, 2021	Single-arm trial	Patients with symptomatic, chronic, and functional TR of grade ≥ moderate, despite medical therapy	30 (80.0)	77.4 ± 7.8	Severe (27.0); massive (20.0); torrential (53.0)	Less than III (30.0); III or IV (70.0)	Atrial fibrillation/flutter (96.7); dyslipidemia (73.3); pulmonary hypertension (73.0); hypertension (62.1); kidney dysfunction (36.7); diabetes (30.0); conduction defects/heart block (26.7); coronary artery disease (13.3); myocardial infarction (13.3); transient ischemic attack (13.3); stroke (6.7); peripheral vascular disease (3.3)	Diuretics (100.0)	30 Days	AD; AA; VC; EROA; RV end-diastolic diameter; RVFAC; sPAP; TVTH; LVEF; IVC diameter; LVSV; NYHA; KCCQ; 6-MWD; TR severity reduction
Gerçek, 2021	Observational study	Patients with TR and acute RCA deformation developed during implantation	14 (77.0)	77.0 ± 8.0	Severe (21.4); massive (7.2); torrential (71.4)	II (7.1); III (78.6); IV (14.3)	Diabetes (42.8); stroke (35.7); coronary artery disease (35.7); COPD (21.4)	-	Discharge	AD; VC; CG; TRD; RV end diastolic diameter; TAPSE; sHR; TR severity reduction
Gray, 2022	Single-arm trial	Patients with symptomatic, chronic, and functional TR of grade ≥ moderate,	37 (75.7)	77.5 ± 7.5	Severe (22.2); massive (19.4); torrential (58.3) *	I (2.7); II (32.4); III (59.5); IV (5.4)	Thyroid disease (100.0); atrial fibrillation/flutter (97.3); pulmonary hypertension (73.0); hypertension (70.3);	Diuretics and anticoagulants (100.0)	Discharge, 30 days, 6 months, 1 year	AD; EROA; VC; RV end-diastolic diameter; TVTH; RAV; inferior VC diameter; LVEF;

		despite optimal medical therapy					dyslipidemia (70.3); renal disease (37.8); chronic anemia (35.1); conduction defects/heart block (29.7); diabetes (27.0); cancer/ malignancy (16.2); transient ischemic attack (13.5); myocardial infarction (10.8); coronary artery disease (10.8); peripheral vascular disease (5.4); stroke (5.4)			TAPSE; TR severity reduction; NYHA; KCCQ; 6-MWD; freedom from heart failure rehospitalization; survival
Körber, 2021	Observational study	Patients with symptomatic, secondary, at least severe TR	60 (61.7)	76 [73 - 82]	Severe (48.3); massive (30.0); torrential (21.7)	I (1.6); II (16.7); III (71.7); IV (10.0)	Atrial fibrillation (90.0); heart failure with preserved ejection fraction (78.0); coronary artery disease (43.4); advanced chronic kidney disease (35.0); diabetes (30.0); RCA stenosis > 50% (30.0); COPD (28.3); right heart infarction (10.0); stroke (16.7); peripheral artery disease (8.0)	Diuretics (98.3); NOACs (46.7); aldosterone antagonists (40.7); oral vitamin K antagonists (40.0)	Discharge, 30 days, 6 months	AD; EROA; RV end-diastolic diameter; vena cava diameter; VC; LVEF; sPAP; TAPSE; TRV; CG; RVVC; SVI; NYHA; TR severity reduction; rehospitalization rate for heart failure; survival
Mattig, 2023	Observational study	Patients with symptomatic, severe to torrential TR, and at high surgical risk	63 (64.0)	81 [77 - 84]	Severe to torrential	II (20.3); III (73.4); IV (6.3)	Atrial fibrillation/flutter (97.0); hypertension (86.0); coronary artery disease (56.0); diabetes (27.0); stroke (17.0); COPD (16.0); peripheral artery disease (8.0); asthma (3.0)	Diuretics (94.0); beta-blockers (86.0); ACEi or sacubitril/ valsartan (78.0); mineralocorticoid receptor antagonists (55.0); SGLT-2i (23.0)	Discharge	AD; RV end-diastolic diameter; LVEF; RAA; TAPSE; RVFAC; TR severity reduction
Nickenig, 2019	Single-arm trial	Patients with symptomatic, chronic, functional, moderate to severe TR and inoperable, and stable medical treatment	30 (73.3)	75.2 ± 6.6	Mild (4.0); moderate (20.0); severe to torrential (76.0) **	I or II (16.7); III or IV (83.3)	Atrial fibrillation/flutter (93.3); hypertension (80.0); congestive heart failure (56.7); chronic renal disease (53.3); dyslipidemia (53.3); pulmonary hypertension (50.0); coronary artery disease (36.7); diabetes (26.7); stroke/transient ischemic attack (16.7); ventricular tachycardia/	-	30 days, 6 months	AD; LVEF; EROA; RV end-diastolic diameter; sPAP; VC; TRV; LVSV; TR severity reduction; NYHA; KCCQ; 6-MWD; EA

						fibrillation (6.7)			
Nickenig, 2021a	Single-arm trial	Patients with symptomatic, chronic, functional TR of grade ≥ moderate and NYHA Functional Class II-IV, despite optimal medical therapy	61 (75.4)	78.6 ± 5.7	Moderate (6.0); severe (26.0); massive (28.0); torrential (40.0) ***	II (14.8); III or IV (85.2)	Atrial fibrillation/flutter (91.8); hypertension (85.0); pulmonary hypertension (45.9); renal disease (42.6); dyslipidemia/hyperlipidemia (34.5); diabetes (27.9); stroke (13.6); conduction defects/heart block (12.3); coronary artery disease (11.9); myocardial infarction (10.3); transient ischemic attack (7.1); peripheral arterial disease (3.4)	Diuretics (100.0); anticoagulants (% unknown)	Discharge, 30 days	AD; EROA; VC; TR severity reduction; RV end-diastolic diameter; RAV; IVC diameter; HVFR; RVFAC; TAPSE; AA; sPAP; TVTH; LVSV; LVEF; CO; NYHA; KCCQ; EQ-5D-5L
Nickenig, 2021b	Single-arm trial	Patients with chronic, functional TR of grade ≥ moderate and NYHA functional Class II-IV, inoperable, symptomatic despite stable medical treatment	30 (73.3)	75.2 ± 6.6	Mild (4.0); moderate (20.0); severe (24.0); massive (16.0); torrential (36.0) **	I or II (16.7); III or IV (83.3)	Atrial fibrillation/flutter (93.3); hypertension (80.0); congestive heart failure (56.7); chronic renal disease (53.3); dyslipidemia (53.3); pulmonary hypertension (50.0); coronary artery disease (36.7); diabetes (26.7); stroke/transient ischemic attack (16.7); ventricular tachycardia/ fibrillation (6.7)	Diuretics (100.0)	Discharge, 1 year, 2 years	AD; EROA; VC; LVEF; sPAP; mid-RV end-diastolic diameter; RVFAC; TAPSE; RAV; TR severity reduction; NYHA Class I-II; 6-MWD; KCCQ; EA; freedom from heart failure rehospitalization; survival
Ochs, 2024	Observational study	Patients with symptomatic TR of grade ≥ severe, at high operative risk	74 (71.6)	78 [73 - 82]	Severe (39.2); massive (29.7); torrential (31.1)	II (4.1); III (90.5); IV (5.4)	Atrial fibrillation (89.2); coronary artery disease (33.8); diabetes (32.4); stroke (21.6); COPD (21.6)	-	Discharge, 30 days, 6 months	TR severity reduction; NYHA Class I-II; survival
Pardo Sanz, 2022	Single-arm trial	Patients with symptomatic,	24 (79.2)	77.8 ± 6.7	Severe (45.8); massive (29.2);	II (33.4); III (58.3);	Atrial fibrillation/flutter (100.0); ascites/oedema	Diuretics (% unknown)	Discharge, 6 months,	AA; EROA; VC; RVFAC; TAPSE;

	chronic, functional TR of grade ≥ severe		torrential (25.0)	IV (8.3)	despite medical treatment (100.0); systolic pulmonary hypertension (50.0); diabetes (25.0); chronic renal disease (4.1); myocardial infarction (8.3); COPD (8.3)	279±246 days^, 1 year	RVEF; TR severity reduction;6-MWD; NYHA Class I-II; SE; freedom from heart failure rehospitalization; survival

Table S2. Details of the risk of bias assessment.

Study name	Selection	Outcome	Total	Score	Risk of bias
Barbieri, 2023	✵✵✵	✵✵✵✵	✵✵✵✵✵✵✵	7/9	Low
Davidson, 2021	✵✵✵	✵✵✵✵	✵✵✵✵✵✵✵	7/9	Low
Gerçek, 2021	✵	✵✵✵✵	✵✵✵✵✵	5/9	Moderate
Gray, 2022	✵✵✵	✵✵✵	✵✵✵✵✵✵	6/9	Moderate
Körber, 2021	✵✵✵✵	✵✵✵	✵✵✵✵✵✵✵	7/9	Low
Mattig, 2023	✵✵✵✵	✵✵✵✵	✵✵✵✵✵✵✵✵	8/9	Low
Nickenig, 2019	✵✵✵	✵✵✵	✵✵✵✵✵✵	6/9	Moderate
Nickenig, 2021a	✵✵✵✵	✵✵	✵✵✵✵✵✵	6/9	Moderate
Nickenig, 2021b	✵✵✵	✵✵✵	✵✵✵✵✵✵	6/9	Moderate
Ochs, 2024	✵✵✵✵	✵✵✵	✵✵✵✵✵✵✵	7/9	Low
Pardo Sanz, 2022	✵✵✵	✵✵✵	✵✵✵✵✵✵	6/9	Moderate

Table S3. Summary of the effects of Cardioband implantation on echocardiographic parameters. The table shows the normal and reference values of echocardiographic parameters used for the diagnosis of tricuspid regurgitation, as reported in the current guidelines. For each parameter, the mean value at baseline and after Cardioband implantation and the mean change after Cardioband implantation are also reported. Abbreviations: CI, confidence interval; EROA, effective regurgitant orifice area; TR, tricuspid regurgitation. Letters: [a] Antunes et al., 2017 (doi: 10.1093/ejcts/ezx279); [b] Hahn, 2019 (doi: 10.1016/j.jcmg.2018.07.033); [c] Hahn, 2022 (doi: 10.1093/ehjci/jeac009), [d] Kossaify, 2018 (doi: 10.4137/CMC.S27462).

Parameter	Normal	Mild TR	Moderate TR	Severe TR	Mean value at baseline [95% CI]	Mean value at baseline (range)	Mean value after Cardioband implantation [95% CI]	Mean value after Cardioband implantation (range)	Mean change after Cardioband implantation [95% CI]
**Annulus diameter**	28 ± 5 mm [a]	-	-	≥ 42 mm [b]	43.85 mm [42.76; 44.94]	41.90-45.50 mm	34.62 mm [32.23; 37.01]	30.0-39.5 mm	-9.31 mm [-11.47; -7.15]
**Vena contracta**	-	< 3.0 mm [c]	3-6.9 mm [c]	≥ 7 mm [c]	14.05 mm [13.07; 15.02]	12.30-15.0 mm	7.58 mm [6.22; 8.94]	5.00-10.00 mm	-6.41 mm [-8.34; -4.49]
**EROA**	-	0.20 cm^2 [c]	0.20-0.29 cm^2 [c]	≥ 0.40 cm^2 [c]	0.87 cm^2 [0.72; 1.01]	0.68-1.14 cm^2	0.35 cm^2 [0.25; 0.46]	0.20-0.55 cm^2	-0.50 cm^2 [-0.72; -0.28]
**Mid right ventricular diameter**	≤ 35 mm	-	-	-	39.14 mm [37.36; 40.92]	37.0-41.0 mm	37.09 mm [36.03; 38.14]	37.0-37.4 mm	-2.14 mm [-3.99; 0.30]
**Base right ventricular diameter**	≤ 42 mm	-	-	-	51.05 mm [47.53; 54.56]	47.0-56.0 mm	46.50 mm [43.41; 49.60]	43.4-52.0 mm	-4.68 mm [-6.14; -3.22]

Table S4. Indirect comparison of the results of our meta-analysis on Cardioband with those of other studies on medical devices for transcatheter tricuspid valve intervention (TTVI). Values are reported as means. Abbreviations: EROA, effective regurgitant orifice area; F-UP, follow-up; LVEF, left ventricular ejection fraction; RVFAC, right ventricular fractional area change; sPAP, systolic pulmonary artery pressure; TAPSE, tricuspid annular plane systolic excursion. Letters: [a] Nickenig et al., 2017 (doi: 10.1161/CIRCULATIONAHA.116.024848; follow-up: 30 days); [b] Mehr et al., 2019 (doi: 10.1016 /j.jcin.2019.04.019; follow-up: 1 year); [c] Nickenig et al., 2019 (doi: 10.1016/S0140-6736(19)32600-5; follow-up: 30 days); [d] Lurz et al., 2023 (doi: 10.1016/j.jacc.2023.05.008; follow-up: 30 days).

Parameter	Cardioband	Mitraclip	TriClip
**Annulus diameter (mm)**	Baseline: 43.9<br>F-UP: 34.6	Baseline: 41.2<br>F-UP: 35.7 [a]	Baseline: 43.3<br>F-UP: 40.6 [c]<br>Baseline: 45.4<br>F-UP: 42.7 [d]
**Vena contracta (mm)**	Baseline: 14.1<br>F-UP: 7.6	Baseline: 11.0<br>F-UP: 6.0 [a]	Baseline: 17.3<br>F-UP: 9.9 [c]<br>Baseline: 8.5<br>F-UP: 5.0 [d]
**EROA (cm²)**	Baseline: 0.9<br>F-UP: 0.4	Baseline: 0.9<br>F-UP: 0.4 [a]	Baseline: 0.7<br>F-UP: 0.4 [c]<br>Baseline: 0.8<br>F-UP: 0.4 [d]
**Right ventricular diameter (mm)**	Mid -> Baseline: 39.1; F-UP: 37.1<br>Base -> Baseline: 51.1; F-UP: 46.5	-	Baseline: 52.7<br>F-UP: 49.3 [c]<br>Baseline: 46.3<br>F-UP: 42.8 [d]
**LVEF (%)**	Baseline: 55.8<br>F-UP: 55.7	Baseline: 46.6<br>F-UP: 48.3 [a]<br>Baseline: 49.0<br>F-UP: 49.6 [b]	Baseline: 59.4<br>F-UP: 60.1 [c]<br>Baseline: 55.8<br>F-UP: 57.7 [d]
**Right atrial volume (ml)**	Baseline: 143.0<br>F-UP: 120.3	Baseline: 107.5<br>F-UP: 98.1 [a]	Baseline: 128.0<br>F-UP: 119.8 [c]<br>Baseline: 151.7<br>F-UP: 136.3 [d]
**TAPSE (mm)**	Baseline: 17.3<br>F-UP: 15.8	Baseline: 16.8<br>F-UP: 17.1 [a]<br>Baseline: 15.8<br>F-UP: 15.9 [b]	Baseline: 14.4<br>F-UP: 14.9 [c]<br>Baseline: 17.0<br>F-UP: 16.9 [d]
**sPAP (mmHg)**	Baseline: 35.3<br>F-UP: 41.1	Baseline: 44.1<br>F-UP: 40.4 [a]<br>Baseline: 43.6<br>F-UP: 39.3 [b]	-
**RVFAC (%)**	Baseline: 39.3<br>F-UP: 36.8	Baseline: 36.5<br>F-UP: 34.6 [a]	Baseline: 35.8<br>F-UP: 36.7 [c]<br>Baseline: 39.4<br>F-UP: 38.9 [d]

Study	Post-implantation Mean	SD	Total	Pre-implantation Mean	SD	Total	MD	95%-CI	Weight
RoB = Low									
Barbieri, 2023	30.00	1.30	65	43.00	1.00	65	-13.00	[-13.40; -12.60]	18.9%
Davidson, 2021	39.50	7.40	27	45.40	4.70	27	-5.90	[-9.21; -2.59]	13.2%
Körber, 2021	32.50	7.00	56	43.00	6.20	56	-10.50	[-12.95; -8.05]	15.3%
Random effects model			148			148	-10.06	[-14.06; -6.06]	47.3%
Heterogeneity: $I^2 = 90\%$, $\tau^2 = 11.1157$, $p < 0.01$									
RoB = Moderate									
Gerçek, 2021	34.00	7.00	14	44.00	7.00	14	-10.00	[-15.19; -4.81]	9.1%
Nickenig, 2021a	36.70	5.00	52	45.50	4.50	52	-8.80	[-10.63; -6.97]	16.7%
Nickenig, 2021b	36.20	4.60	22	41.90	4.60	26	-5.70	[-8.31; -3.09]	14.9%
Pardo Sanz, 2022	34.10	7.70	24	44.50	5.60	24	-10.40	[-14.21; -6.59]	12.0%
Random effects model			112			116	-8.36	[-10.49; -6.22]	52.7%
Heterogeneity: $I^2 = 47\%$, $\tau^2 = 2.2555$, $p = 0.13$									
**Random effects model**			**260**			**264**	**-9.31**	**[-11.47; -7.15]**	**100.0%**

Heterogeneity: $I^2 = 91\%$, $\tau^2 = 6.4124$, $p < 0.01$
Test for subgroup differences: $\chi^2_1 = 0.54$, df = 1 ($p = 0.46$)

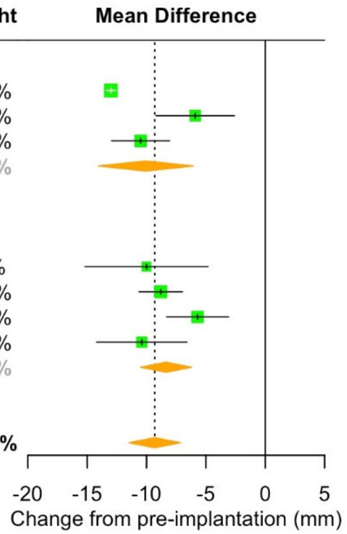

Figure S1. Forest plot of mean difference (MD) with 95% Confidence Interval (CI) of annulus diameter (mm) between post-implantation and pre-implantation of Cardioband in patients with tricuspid regurgitation, stratified by risk of bias.

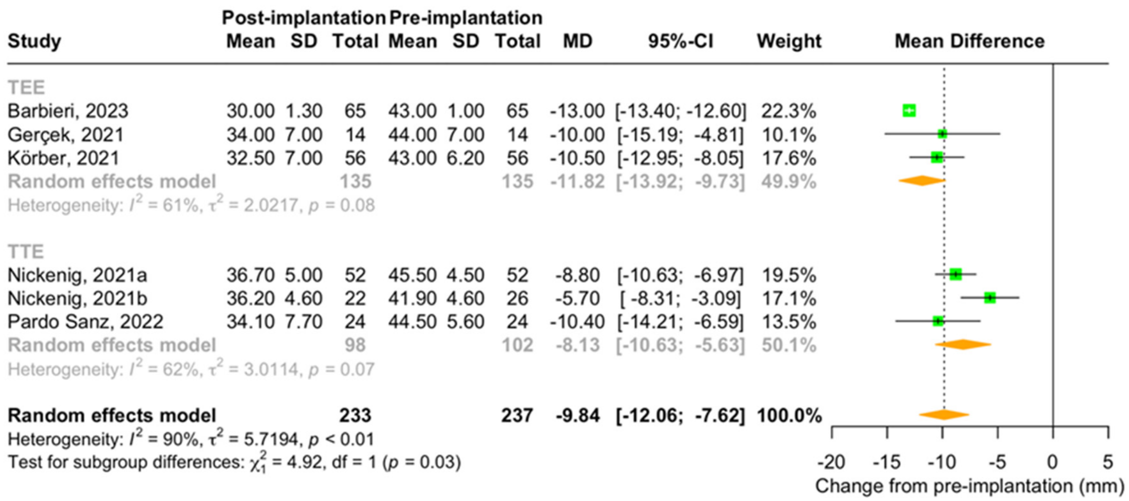

Figure S2. Forest plot of mean difference (MD) with 95% Confidence Interval (CI) of annulus diameter (mm) between post-implantation and pre-implantation of Cardioband in patients with tricuspid regurgitation, stratified by echocardiographic technique (TEE: transesophageal echocardiography; TTE: transthoracic echocardiography).

Study	Post-implantation Mean	Post-implantation SD	Post-implantation Total	Pre-implantation Mean	Pre-implantation SD	Pre-implantation Total	MD	95%-CI	Weight
**Not specified**									
Barbieri, 2023	30.00	1.30	65	43.00	1.00	65	-13.00	[-13.40; -12.60]	18.9%
Gerçek, 2021	34.00	7.00	14	44.00	7.00	14	-10.00	[-15.19; -4.81]	9.1%
Random effects model			79			79	-12.66	[-14.52; -10.80]	28.0%
Heterogeneity: $I^2 = 22\%$, $\tau^2 = 0.9793$, $p = 0.26$									
**End-diastolic septolateral AD**									
Davidson, 2021	39.50	7.40	27	45.40	4.70	27	-5.90	[-9.21; -2.59]	13.2%
Körber, 2021	32.50	7.00	56	43.00	6.20	56	-10.50	[-12.95; -8.05]	15.3%
Nickenig, 2021a	36.70	5.00	52	45.50	4.50	52	-8.80	[-10.63; -6.97]	16.7%
Nickenig, 2021b	36.20	4.60	22	41.90	4.60	26	-5.70	[-8.31; -3.09]	14.9%
Pardo Sanz, 2022	34.10	7.70	24	44.50	5.60	24	-10.40	[-14.21; -6.59]	12.0%
Random effects model			181			185	-8.27	[-10.24; -6.29]	72.0%
Heterogeneity: $I^2 = 61\%$, $\tau^2 = 3.0882$, $p = 0.03$									
**Random effects model**			**260**			**264**	**-9.31**	**[-11.47; -7.15]**	**100.0%**
Heterogeneity: $I^2 = 91\%$, $\tau^2 = 6.4124$, $p < 0.01$									
Test for subgroup differences: $\chi^2_1 = 10.07$, df = 1 ($p < 0.01$)									

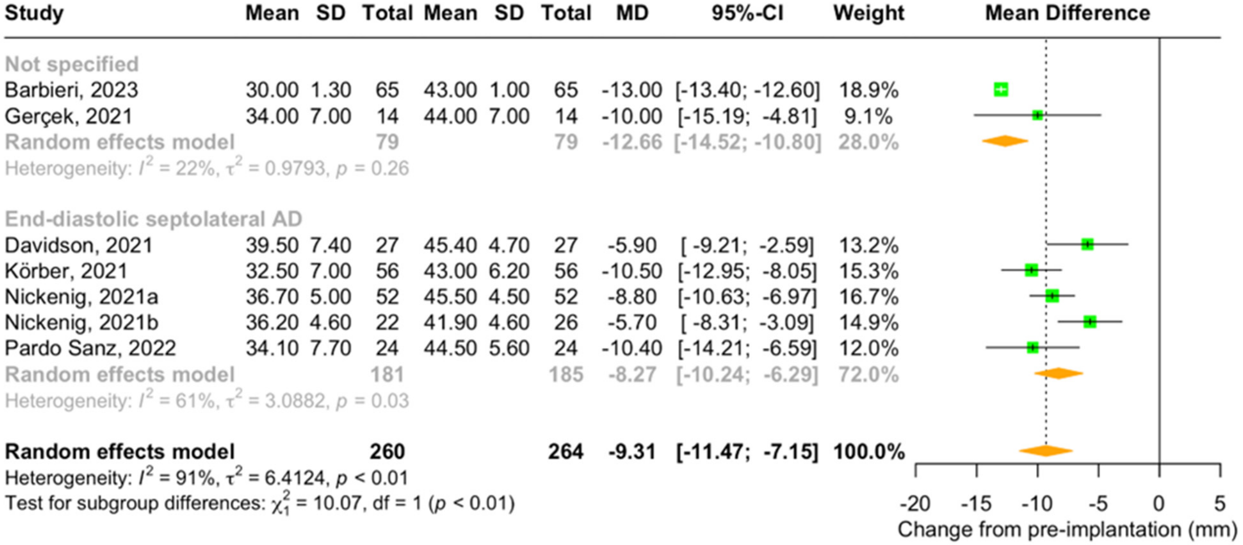

Figure S3. Forest plot of mean difference (MD) with 95% Confidence Interval (CI) of annulus diameter (mm) between post-implantation and pre-implantation of Cardioband in patients with tricuspid regurgitation, stratified by the "phase" at which the parameter was measured (i.e., end-diastolic septolateral annulus diameter or not specified).

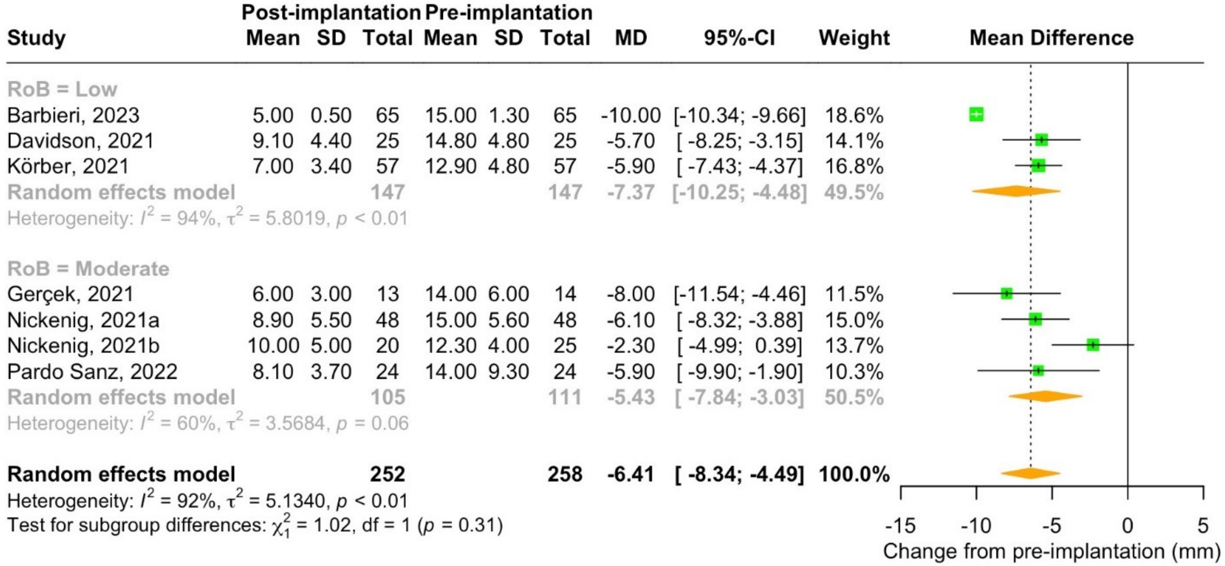

Figure S4. Forest plot of mean difference (MD) with 95% Confidence Interval (CI) of vena contracta (mm) between post-implantation and pre-implantation of Cardioband in patients with tricuspid regurgitation, stratified by risk of bias.

Study	Post-implantation Mean	SD	Total	Pre-implantation Mean	SD	Total	MD	95%-CI	Weight
TEE									
Barbieri, 2023	5.00	0.50	65	15.00	1.30	65	-10.00	[-10.34; -9.66]	21.0%
Gerçek, 2021	6.00	3.00	13	14.00	6.00	14	-8.00	[-11.54; -4.46]	13.7%
Random effects model			78			79	-9.81	[-10.96; -8.65]	34.7%
Heterogeneity: $I^2 = 18\%$, $\tau^2 = 0.3532$, $p = 0.27$									
TTE									
Körber, 2021	7.00	3.40	57	12.90	4.80	57	-5.90	[-7.43; -4.37]	19.2%
Nickenig, 2021a	8.90	5.50	48	15.00	5.60	48	-6.10	[-8.32; -3.88]	17.4%
Nickenig, 2021b	10.00	5.00	20	12.30	4.00	25	-2.30	[-4.99; 0.39]	16.1%
Pardo Sanz, 2022	8.10	3.70	24	14.00	9.30	24	-5.90	[-9.90; -1.90]	12.5%
Random effects model			149			154	-5.14	[-6.88; -3.39]	65.3%
Heterogeneity: $I^2 = 49\%$, $\tau^2 = 1.5897$, $p = 0.12$									
**Random effects model**			**227**			**233**	**-6.50**	**[-8.73; -4.28]**	**100.0%**
Heterogeneity: $I^2 = 93\%$, $\tau^2 = 6.0873$, $p < 0.01$									
Test for subgroup differences: $\chi^2_1 = 19.19$, df = 1 ($p < 0.01$)									

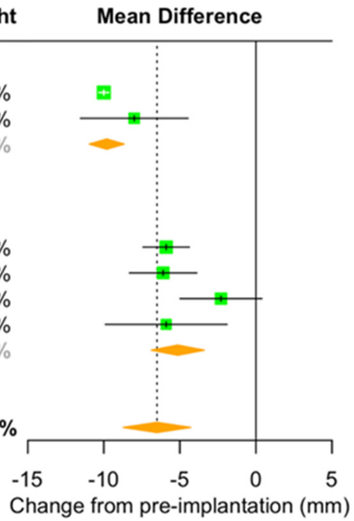

Figure S5. Forest plot of mean difference (MD) with 95% Confidence Interval (CI) of vena contracta (mm) between post-implantation and pre-implantation of Cardioband in patients with tricuspid regurgitation, stratified by echocardiographic technique (TEE: transesophageal echocardiography; TTE: transthoracic echocardiography).

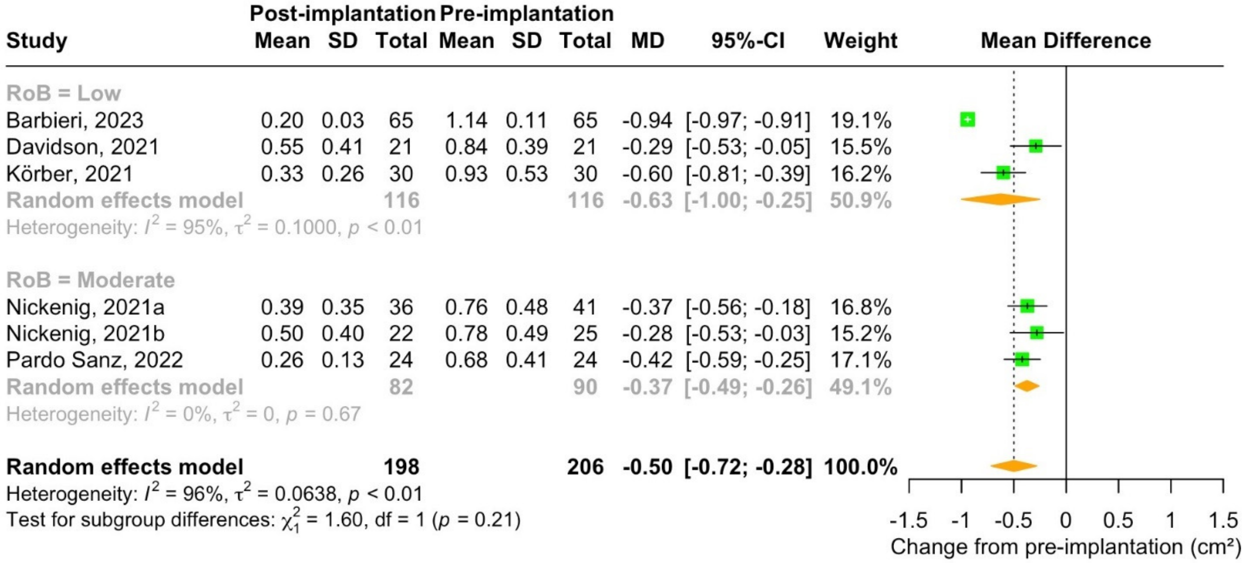

Figure S6. Forest plot of mean difference (MD) with 95% Confidence Interval (CI) of effective regurgitant orifice area (EROA; cm²) between post-implantation and pre-implantation of Cardioband in patients with tricuspid regurgitation, stratified by risk of bias.

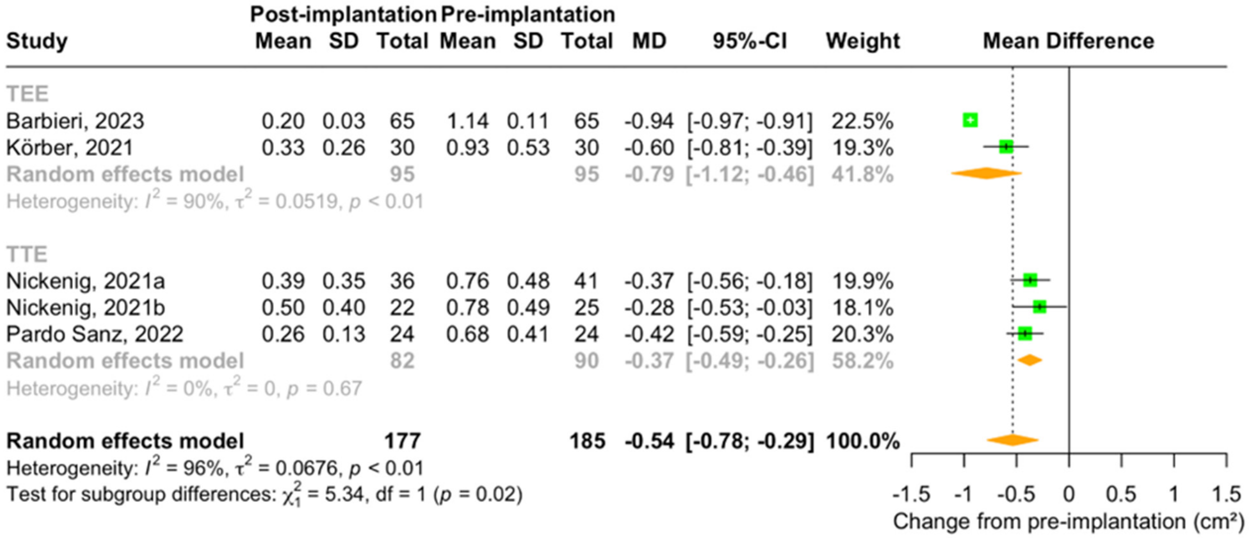

Figure S7. Forest plot of mean difference (MD) with 95% Confidence Interval (CI) of effective regurgitant orifice area (EROA; cm²) between post-implantation and pre-implantation of Cardioband in patients with tricuspid regurgitation, stratified by echocardiographic technique (TEE: transesophageal echocardiography; TTE: transthoracic echocardiography).

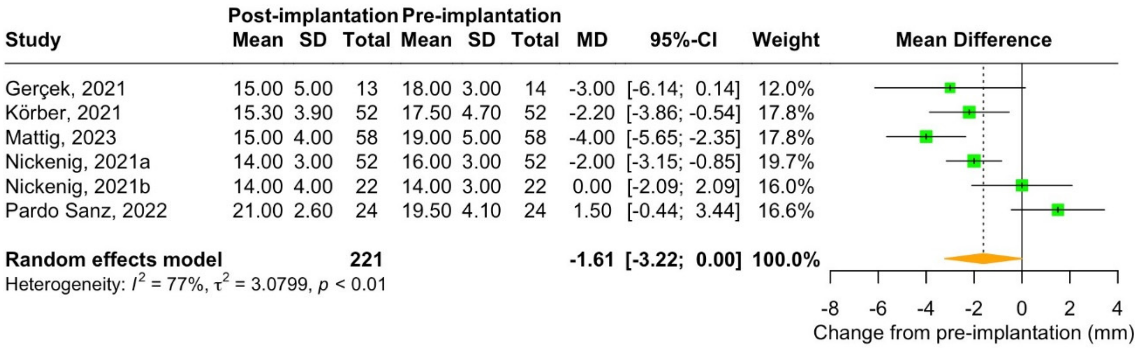

Figure S8. Forest plot of mean difference (MD) with 95% Confidence Interval (CI) of tricuspid annular plane systolic excursion (TAPSE; mm) between post-implantation and pre-implantation of Cardioband in patients with tricuspid regurgitation.

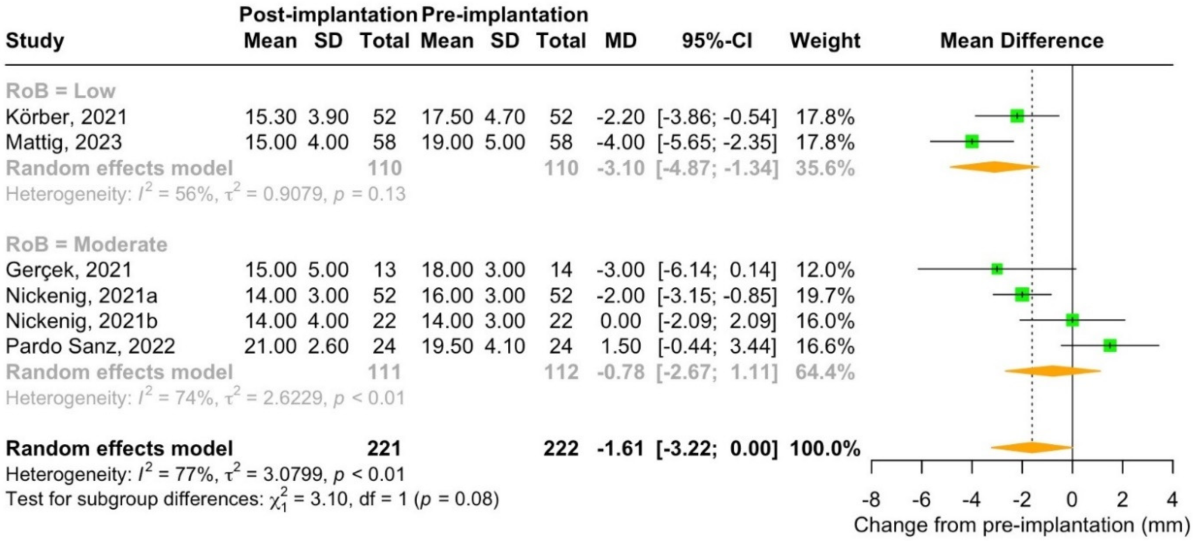

Figure S9. Forest plot of mean difference (MD) with 95% Confidence Interval (CI) of tricuspid annular plane systolic excursion (TAPSE; mm) between post-implantation and pre-implantation of Cardioband in patients with tricuspid regurgitation, stratified by risk of bias.

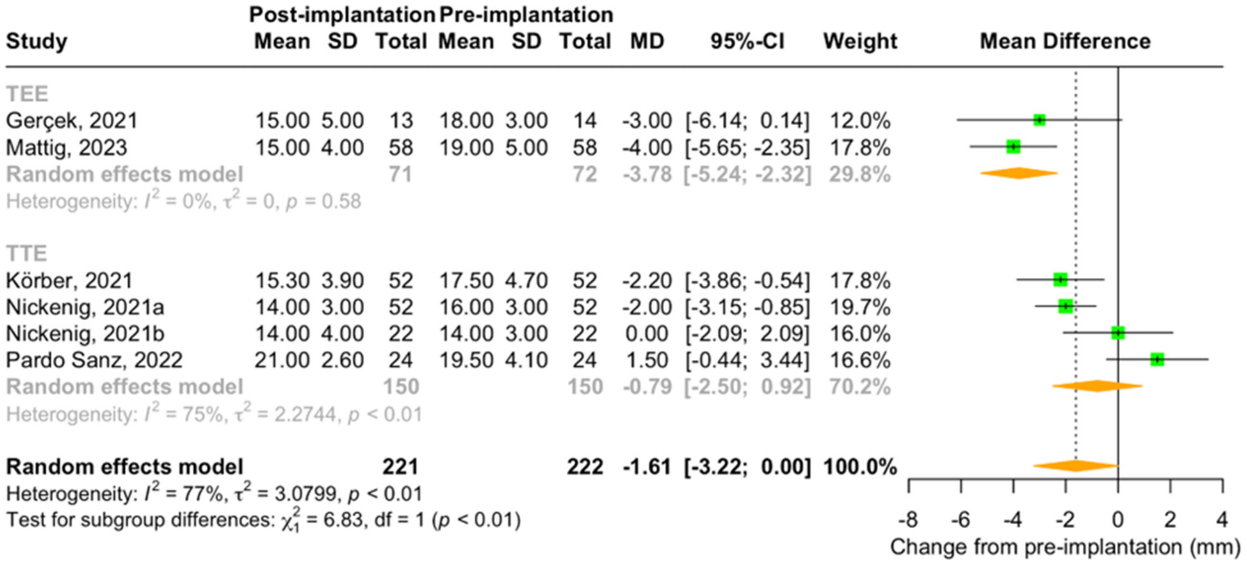

Figure S10. Forest plot of mean difference (MD) with 95% Confidence Interval (CI) of tricuspid annular plane systolic excursion (TAPSE; mm) between post-implantation and pre-implantation of Cardioband in patients with tricuspid regurgitation, stratified by echocardiographic technique (TEE: transesophageal echocardiography; TTE: transthoracic echocardiography).

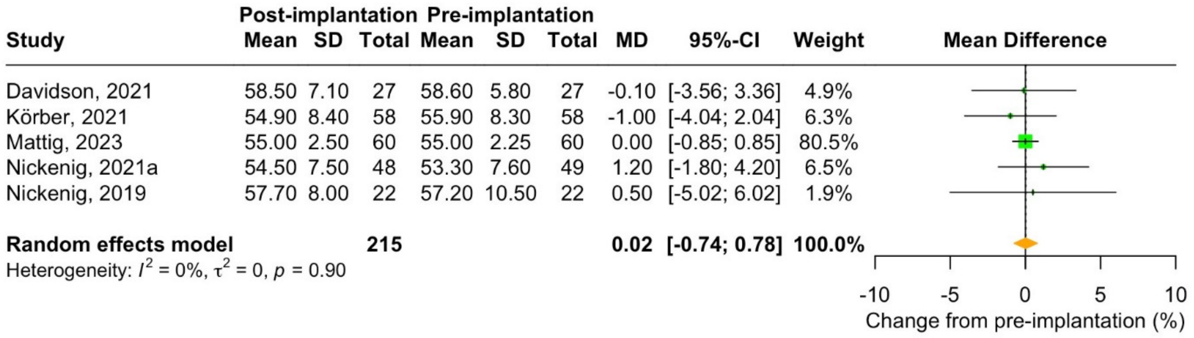

Figure S11. Forest plot of mean difference (MD) with 95% Confidence Interval (CI) of left ventricular ejection fraction (LVEF; %) between post-implantation and pre-implantation of Cardioband in patients with tricuspid regurgitation.

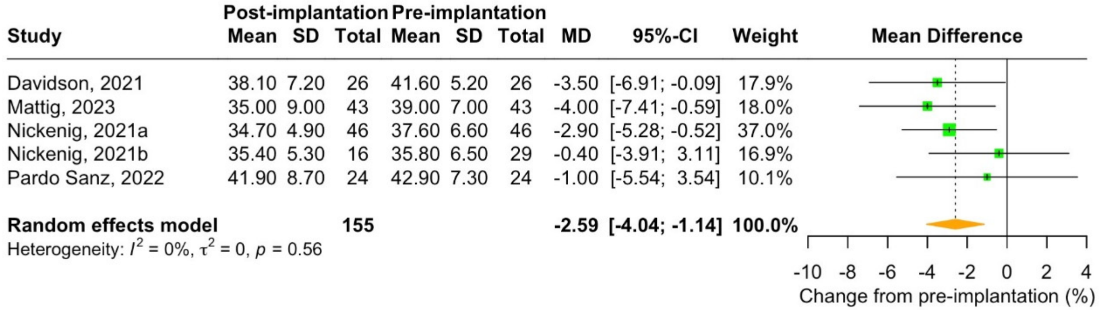

Figure S12. Forest plot of mean difference (MD) with 95% Confidence Interval (CI) of right ventricular fractional area change (RVFAC; %) between post-implantation and pre-implantation of Cardioband in patients with tricuspid regurgitation.

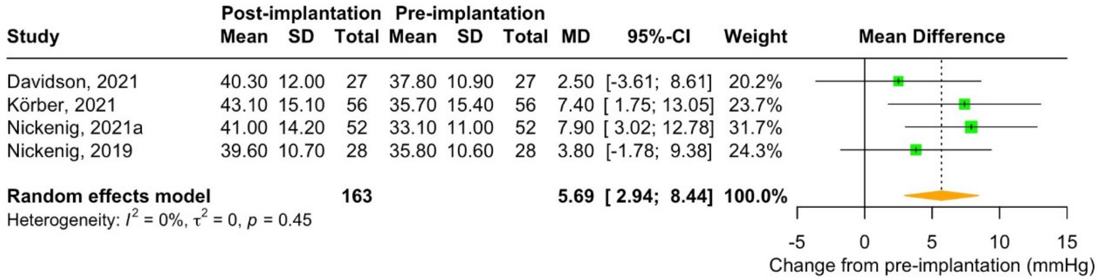

Figure S13. Forest plot of mean difference (MD) with 95% Confidence Interval (CI) of systolic pulmonary artery pressure (sPAP; mmHg) between post-implantation and pre-implantation of Cardioband in patients with tricuspid regurgitation.

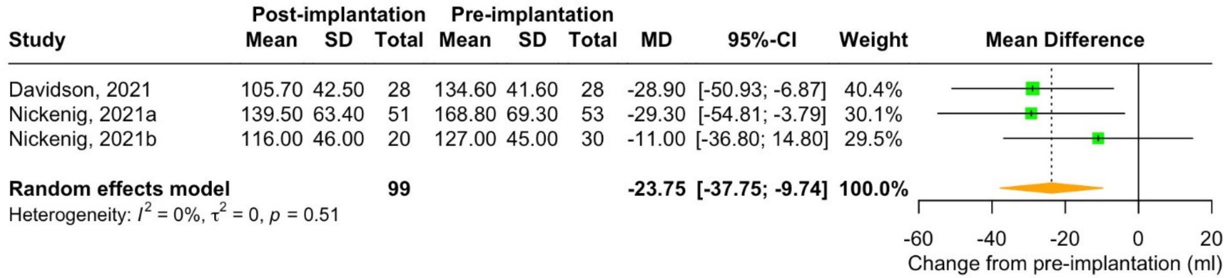

Figure S14. Forest plot of mean difference (MD) with 95% Confidence Interval (CI) of right atrial volume (ml) between post-implantation and pre-implantation of Cardioband in patients with tricuspid regurgitation.

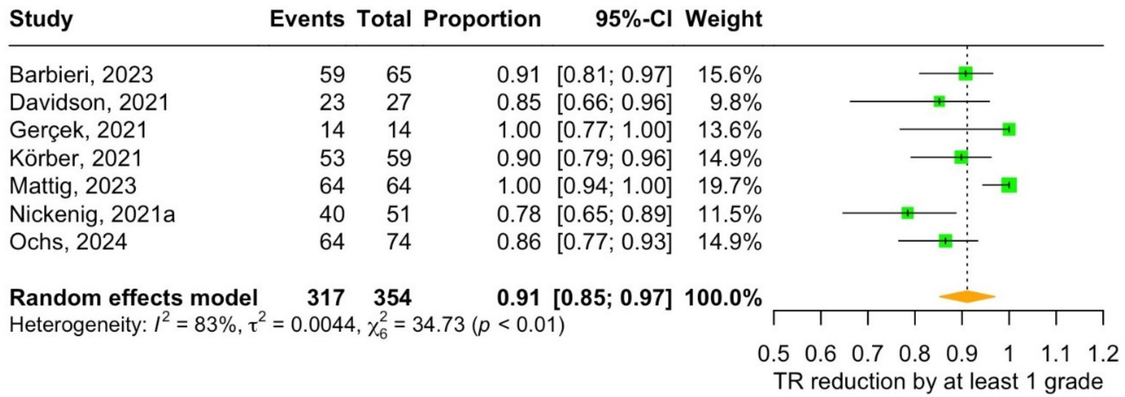

Figure S15. Forest plot of proportions, with 95% Confidence Interval (CI), of patients with tricuspid regurgitation reduction of at least one grade after Cardioband implantation.

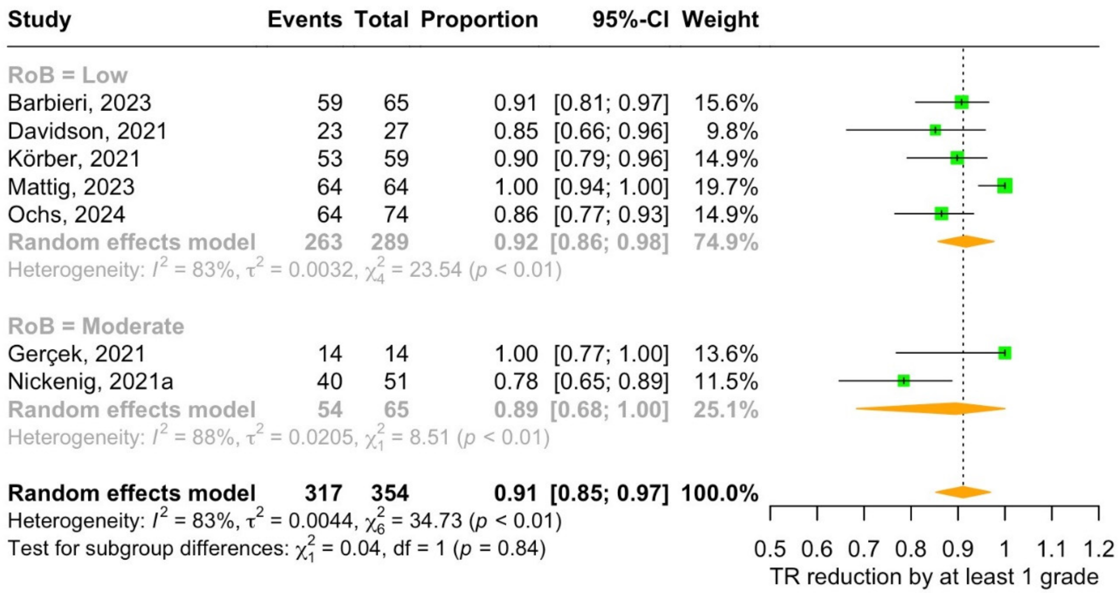

Figure S16. Forest plot of proportions, with 95% Confidence Interval (CI), of patients with tricuspid regurgitation reduction of at least one grade after Cardioband implantation, stratified by risk of bias.

Study	Events	Total	Proportion	95%-CI	Weight
TEE					
Barbieri, 2023	59	65	0.91	[0.81; 0.97]	17.3%
Gerçek, 2021	14	14	1.00	[0.77; 1.00]	15.1%
Ochs, 2024	64	74	0.86	[0.77; 0.93]	16.5%
Random effects model	137	153	0.92	[0.85; 0.99]	49.0%
Heterogeneity: $I^2 = 60\%$, $\tau^2 = 0.0026$, $\chi^2_2 = 4.99$ ($p = 0.08$)					
TTE					
Körber, 2021	53	59	0.90	[0.79; 0.96]	16.6%
Mattig, 2023	64	64	1.00	[0.94; 1.00]	21.5%
Nickenig, 2021a	40	51	0.78	[0.65; 0.89]	12.9%
Random effects model	157	174	0.90	[0.78; 1.00]	51.0%
Heterogeneity: $I^2 = 89\%$, $\tau^2 = 0.0099$, $\chi^2_2 = 18.9$ ($p < 0.01$)					
**Random effects model**	**294**	**327**	**0.92**	**[0.85; 0.98]**	**100.0%**
Heterogeneity: $I^2 = 84\%$, $\tau^2 = 0.0047$, $\chi^2_5 = 31.57$ ($p < 0.01$)					
Test for subgroup differences: $\chi^2_1 = 0.05$, df = 1 ($p = 0.82$)					

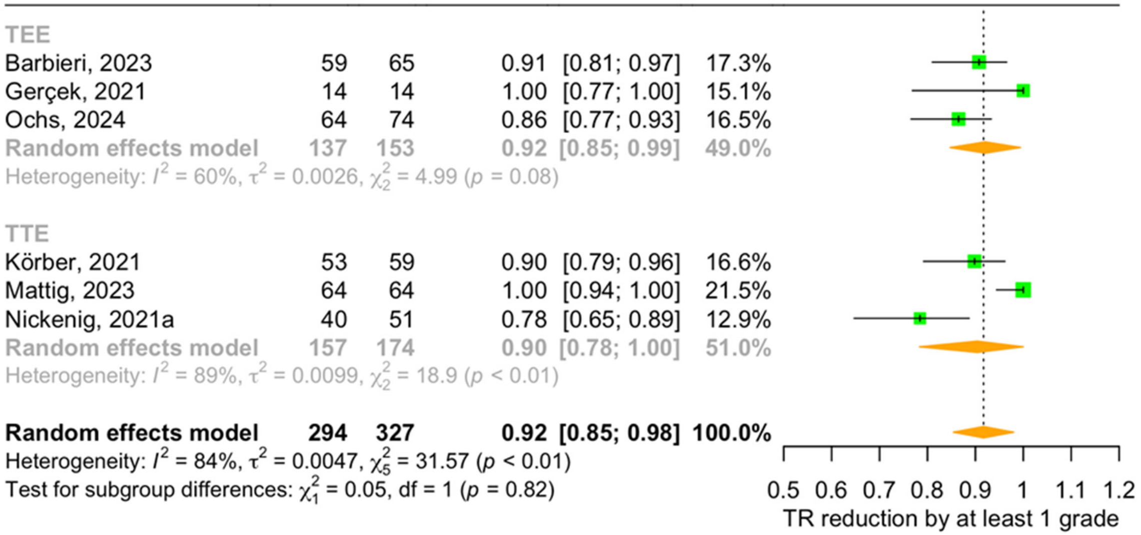

Figure S17. Forest plot of proportions, with 95% Confidence Interval (CI), of patients with tricuspid regurgitation reduction of at least one grade after Cardioband implantation, stratified by echocardiographic technique (TEE: transesophageal echocardiography; TTE: transthoracic echocardiography).

Study	Events	Total	Proportion	95%-CI	Weight
Barbieri, 2023	51	65	0.78	[0.67; 0.88]	12.4%
Gerçek, 2021	10	14	0.71	[0.42; 0.92]	8.7%
Gray, 2022	16	36	0.44	[0.28; 0.62]	10.8%
Körber, 2021	36	59	0.61	[0.47; 0.73]	11.8%
Mattig, 2023	48	60	0.80	[0.68; 0.89]	12.3%
Nickenig, 2021a	29	54	0.54	[0.40; 0.67]	11.6%
Nickenig, 2021b	5	22	0.23	[0.08; 0.45]	10.4%
Ochs, 2024	49	74	0.66	[0.54; 0.77]	12.2%
Pardo Sanz, 2022	15	24	0.62	[0.41; 0.81]	9.9%
**Random effects model**	**259**	**408**	**0.61**	**[0.49; 0.72]**	**100.0%**

Heterogeneity: $I^2 = 83\%$, $\tau^2 = 0.0258$, $\chi^2_8 = 47.51$ ($p < 0.01$)

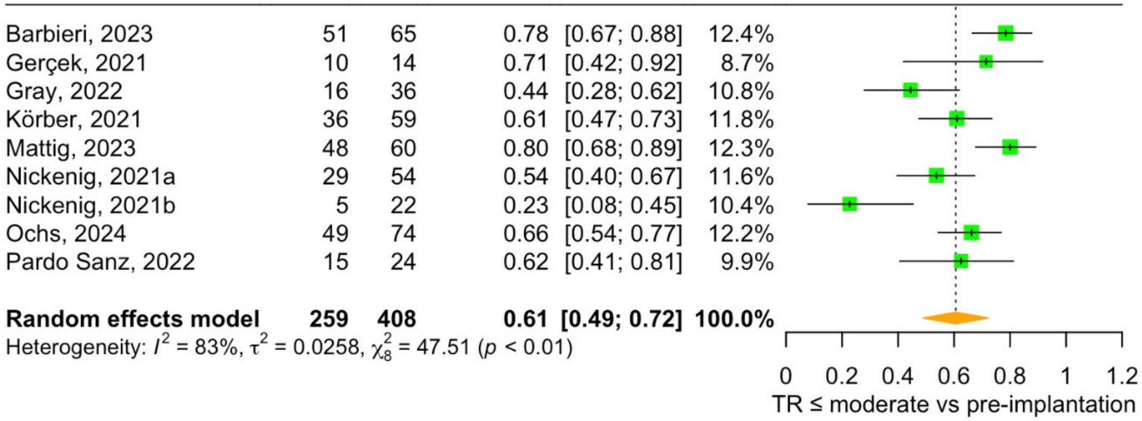

Figure S18. Forest plot of proportions, with 95% Confidence Interval (CI), of patients with tricuspid regurgitation reduction to at least moderate grade after Cardioband implantation.

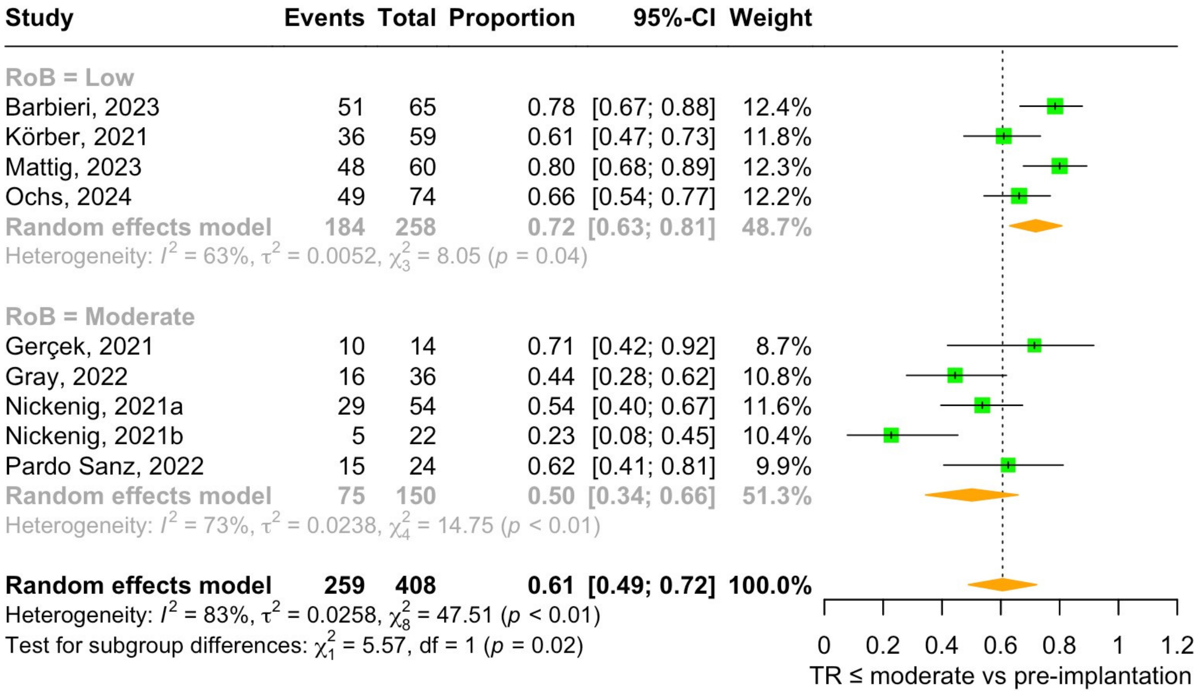

Figure S19. Forest plot of proportions, with 95% Confidence Interval (CI), of patients with tricuspid regurgitation reduction to at least moderate grade after Cardioband implantation, stratified by risk of bias.

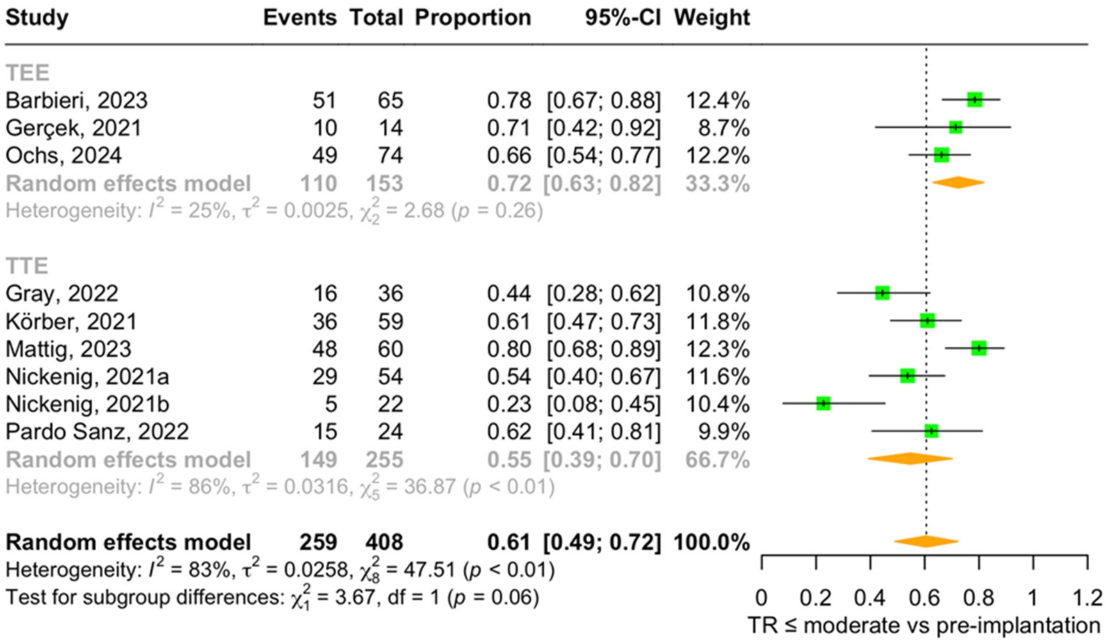

Figure S20. Forest plot of proportions, with 95% Confidence Interval (CI), of patients with tricuspid regurgitation reduction to at least moderate grade after Cardioband implantation, stratified by echocardiographic technique (TEE: transesophageal echocardiography; TTE: transthoracic echocardiography).

Study	Events	Total	Proportion	95%-CI	Weight
Davidson,2021	12	28	0.43	[0.24; 0.63]	14.4%
Körber, 2021	29	48	0.60	[0.45; 0.74]	17.3%
Nickenig, 2021a	28	50	0.56	[0.41; 0.70]	17.3%
Nickenig, 2019	18	23	0.78	[0.56; 0.93]	15.3%
Ochs, 2024	44	74	0.59	[0.47; 0.71]	19.0%
Pardo Sanz, 2022	20	24	0.83	[0.63; 0.95]	16.6%
**Random effects model**	**151**	**247**	**0.63**	**[0.52; 0.75]**	**100.0%**

Heterogeneity: $I^2 = 70\%$, $\tau^2 = 0.0141$, $\chi^2_5 = 16.44$ ($p < 0.01$)

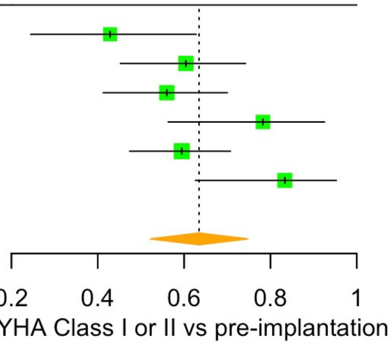

Figure S21. Forest plot of proportions, with 95% Confidence Interval (CI), of patients in New York Heart Association (NYHA) Functional Class I or II after Cardioband implantation.

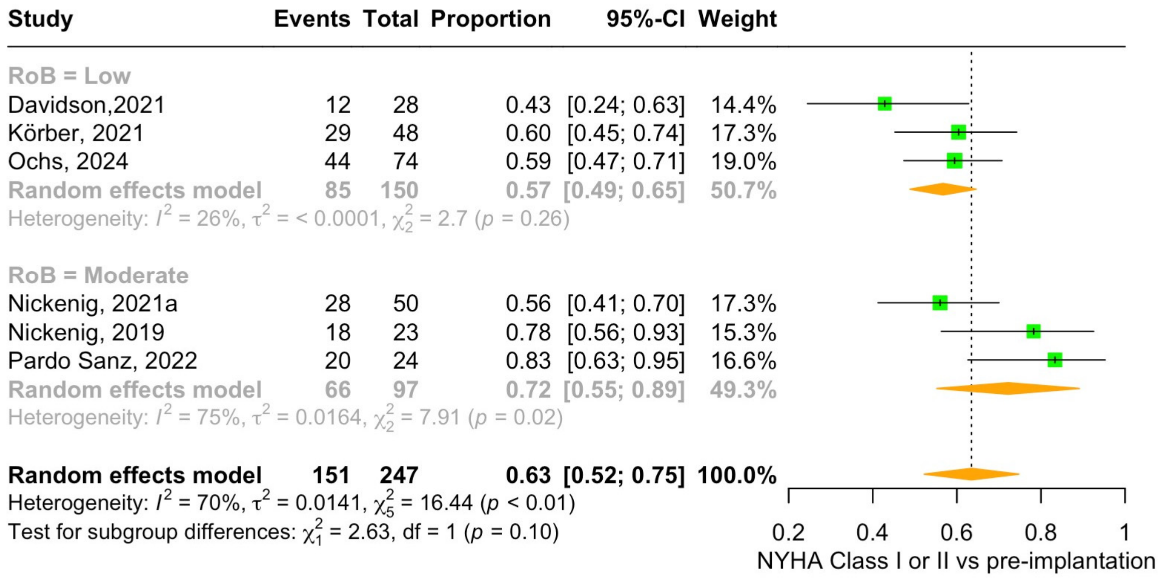

Figure S22. Forest plot of proportions, with 95% Confidence Interval (CI), of patients in New York Heart Association (NYHA) Functional Class I or II after Cardioband implantation, stratified by risk of bias.

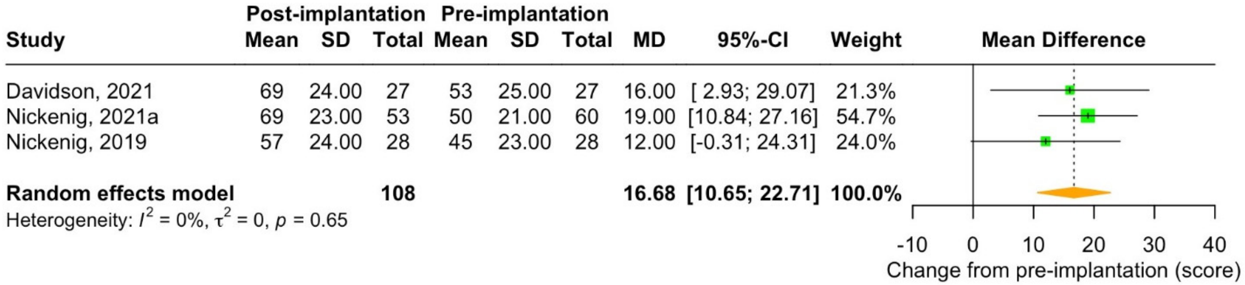

Figure S23. Forest plot of mean difference (MD) with 95% Confidence Interval (CI) of Kansas City Cardiomiopathy Questionnaire (KCCQ) score between post-implantation and pre-implantation of Cardioband in patients with tricuspid regurgitation.

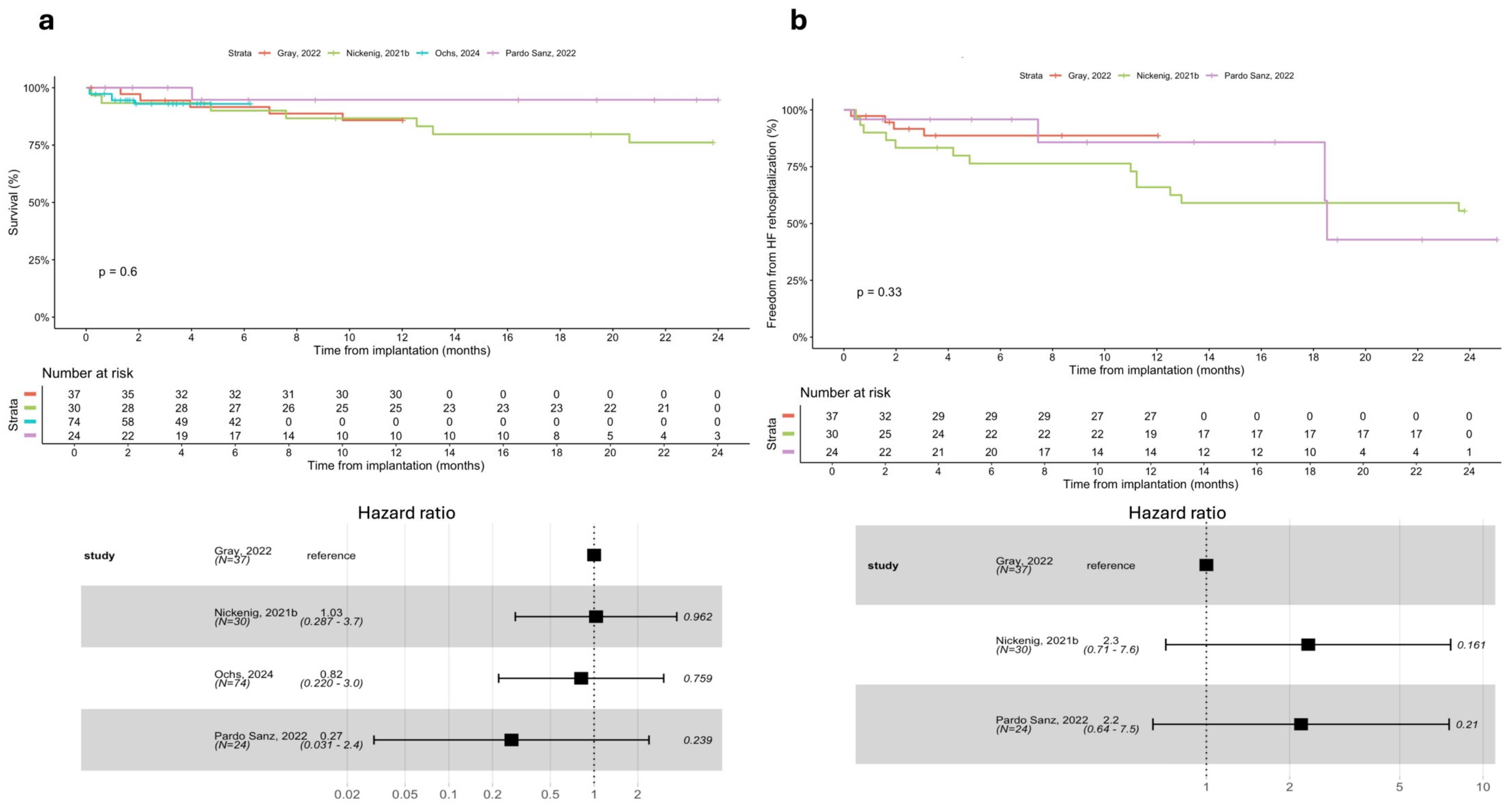

Figure S24. Survival (a) and freedom from heart failure (HF) rehospitalization (b) curves of reconstructed individual patient data (IPD). Forest plots show the hazard ratios (HRs), with 95% Confidence Interval (CI), calculated with the Cox proportional hazards model.

PRISMA CHECKLIST 2020

Section and Topic	Item #	Checklist item	Location where item is reported
**TITLE**			
Title	1	Identify the report as a systematic review.	Title (pag. 1)
**ABSTRACT**			
Abstract	2	See the PRISMA 2020 for Abstracts checklist.	Pag.1, lines 12-30
**INTRODUCTION**			
Rationale	3	Describe the rationale for the review in the context of existing knowledge.	Pag.1-2, lines 34-78
Objectives	4	Provide an explicit statement of the objective(s) or question(s) the review addresses.	Pag.2, lines 79-84
**METHODS**			
Eligibility criteria	5	Specify the inclusion and exclusion criteria for the review and how studies were grouped for the syntheses.	Pag.3, lines 104-117
Information sources	6	Specify all databases, registers, websites, organisations, reference lists and other sources searched or consulted to identify studies. Specify the date when each source was last searched or consulted.	Pag.2-3, lines 89-94
Search strategy	7	Present the full search strategies for all databases, registers and websites, including any filters and limits used.	Supplementary Material S1
Selection process	8	Specify the methods used to decide whether a study met the inclusion criteria of the review, including how many reviewers screened each record and each report retrieved, whether they worked independently, and if applicable, details of automation tools used in the process.	Pag.3, lines 95-103
Data collection process	9	Specify the methods used to collect data from reports, including how many reviewers collected data from each report, whether they worked independently, any processes for obtaining or confirming data from study investigators, and if applicable, details of automation tools used in the process.	Pag.3, lines 126-127
Data items	10a	List and define all outcomes for which data were sought. Specify whether all results that were compatible with each outcome domain in each study were sought (e.g. for all measures, time points, analyses), and if not, the methods used to decide which results to collect.	Pag.3, lines 118-127
	10b	List and define all other variables for which data were sought (e.g. participant and intervention characteristics, funding sources). Describe any assumptions made about any missing or unclear information.	Pag.3, lines 118-127
Study risk of bias assessment	11	Specify the methods used to assess risk of bias in the included studies, including details of the tool(s) used, how many reviewers assessed each study and whether they worked independently, and if applicable, details of automation tools used in the process.	Pag.3, lines 128-141
Effect measures	12	Specify for each outcome the effect measure(s) (e.g. risk ratio, mean difference) used in the synthesis or presentation of results.	Pag.4-5, lines 144-202
Synthesis methods	13a	Describe the processes used to decide which studies were eligible for each synthesis (e.g. tabulating the study intervention characteristics and comparing against the planned groups for each synthesis (item #5)).	Pag.4, lines 145-146, Table S1

Section and Topic	Item #	Checklist item	Location where item is reported
	13b	Describe any methods required to prepare the data for presentation or synthesis, such as handling of missing summary statistics, or data conversions.	Pag.4, lines 147-159
	13c	Describe any methods used to tabulate or visually display results of individual studies and syntheses.	Pag.4, lines 145-146
	13d	Describe any methods used to synthesize results and provide a rationale for the choice(s). If meta-analysis was performed, describe the model(s), method(s) to identify the presence and extent of statistical heterogeneity, and software package(s) used.	Pag.4-5, lines 144-172
	13e	Describe any methods used to explore possible causes of heterogeneity among study results (e.g. subgroup analysis, meta-regression).	Pag.4, lines 170-172
	13f	Describe any sensitivity analyses conducted to assess robustness of the synthesized results.	Pag.4, lines 170-172
Reporting bias assessment	14	Describe any methods used to assess risk of bias due to missing results in a synthesis (arising from reporting biases).	Pag.7, lines 273-276
Certainty assessment	15	Describe any methods used to assess certainty (or confidence) in the body of evidence for an outcome.	Pag.3, lines 128-141
**RESULTS**			
Study selection	16a	Describe the results of the search and selection process, from the number of records identified in the search to the number of studies included in the review, ideally using a flow diagram.	Pag.5, lines 205-208, Figure 1
	16b	Cite studies that might appear to meet the inclusion criteria, but which were excluded, and explain why they were excluded.	Pag.5, lines 209-213
Study characteristics	17	Cite each included study and present its characteristics.	Pag.6, lines 216-268, Table S1
Risk of bias in studies	18	Present assessments of risk of bias for each included study.	Pag.7, lines 269-276, Figure 2, Table S2
Results of individual studies	19	For all outcomes, present, for each study: (a) summary statistics for each group (where appropriate) and (b) an effect estimate and its precision (e.g. confidence/credible interval), ideally using structured tables or plots.	Pag. 7-17, Figures 3-9, and Tables 1-3, Figures S8, S11-S15, S18, S21, S23-S24 and Table S3
Results of syntheses	20a	For each synthesis, briefly summarise the characteristics and risk of bias among contributing studies.	Pag. 7-14, lines 296-297, 335-336, 367-368, 422-424, 446-

Section and Topic	Item #	Checklist item	Location where item is reported
			448, 451-455, 471-474, Figures S1, S4, S6, S9, S16, S19, S22
	20b	Present results of all statistical syntheses conducted. If meta-analysis was done, present for each the summary estimate and its precision (e.g. confidence/credible interval) and measures of statistical heterogeneity. If comparing groups, describe the direction of the effect.	Pag. 7-18, lines 283-286, 323-326, 352-355, 392-400, 414,-421, 443-446, 449-451, 468-471, 476-479, Figures 3-7, and Table 1, Figures S8, S11-S15, S18, S21, S23 and Table S3
	20c	Present results of all investigations of possible causes of heterogeneity among study results.	Pag. 7-14, lines 296-307, 335-341, 367-373, 422-424, 427-433, 446-448, 451-457, 471-474, Figures S1-S7, S9, S10, S16, S19, S20, S22
	20d	Present results of all sensitivity analyses conducted to assess the robustness of the synthesized results.	Pag.7-12, lines 291-296, 330-335, 358-365, 400-402, 424-427, Table S3
Reporting biases	21	Present assessments of risk of bias due to missing results (arising from reporting biases) for each synthesis assessed.	Pag.7, Figure 2, Table S2
Certainty of evidence	22	Present assessments of certainty (or confidence) in the body of evidence for each outcome assessed.	Pag.7, lines 269-276, Figure 2, Table S2
**DISCUSSION**			
Discussion	23a	Provide a general interpretation of the results in the context of other evidence.	Pag. 18-20,

Section and Topic	Item #	Checklist item	Location where item is reported
			lines 551-693
	23b	Discuss any limitations of the evidence included in the review.	Pag.18-20, lines 585-586, 655-657, 662-693
	23c	Discuss any limitations of the review processes used.	Pag.20-21, lines 703-711
	23d	Discuss implications of the results for practice, policy, and future research.	Pag.21, lines 711-715
**OTHER INFORMATION**			
Registration and protocol	24a	Provide registration information for the review, including register name and registration number, or state that the review was not registered.	Pag. 2, lines 86-88
	24b	Indicate where the review protocol can be accessed, or state that a protocol was not prepared.	Pag. 2, line 88
	24c	Describe and explain any amendments to information provided at registration or in the protocol.	Not applicable
Support	25	Describe sources of financial or non-financial support for the review, and the role of the funders or sponsors in the review.	Pag. 22, line 789
Competing interests	26	Declare any competing interests of review authors.	Pag. 22, line 793
Availability of data, code and other materials	27	Report which of the following are publicly available and where they can be found: template data collection forms; data extracted from included studies; data used for all analyses; analytic code; any other materials used in the review.	Pag. 22, line 792
